# Snail Augments Nuclear Deformability to Promote Lymph Node Metastasis of Head and Neck Squamous Cell Carcinoma

**DOI:** 10.3389/fcell.2022.809738

**Published:** 2022-02-21

**Authors:** Yin-Quan Chen, Chen-Yu Hung, Ming-Tzo Wei, Jean-Cheng Kuo, Muh-Hwa Yang, Han-Ying Cheng, Arthur Chiou

**Affiliations:** ^1^ Cancer Progression Research Center, National Yang Ming Chiao Tung University, Taipei, Taiwan; ^2^ Institute of Biochemistry and Molecular Biology, National Yang Ming Chiao Tung University, Taipei, Taiwan; ^3^ Department of Chemical and Biological Engineering, Princeton University, Princeton, NJ, United States; ^4^ Institute of Clinical Medicine, National Yang Ming Chiao Tung University, Taipei, Taiwan; ^5^ Division of Medical Oncology, Department of Oncology, Taipei Veterans General Hospital, Taipei, Taiwan; ^6^ Institute of Biophotonics, National Yang Ming Chiao Tung University, Taipei, Taiwan

**Keywords:** lymph node metastasis, snail, cell mechanics, invasion, nuclear strain

## Abstract

Up to 50% of head and neck squamous cell carcinoma (HNSCC) patients have lymph node (LN) metastasis, resulting in poor survival rate. Numerous studies have supported the notion that the alterations of gene expression and mechanical properties of cancer cells play an important role in cancer metastasis. However, which genes and how they regulate the biomechanical properties of HNSCC cells to promote LN metastasis remains elusive. In this study, we used an LN-metastatic mouse model *in vivo* to generate an LN-metastatic head and neck squamous cell carcinoma cell line and compared the differences in the biomolecular and biomechanical properties of LN-metastatic and non-metastatic cells. Our results showed that LN-metastatic cells had a higher level of Snail expression compared to non-LN-metastatic cells. The higher Snail expression promoted the cellular invasion capability in confined environments, mainly by increasing the longitudinal strain of the cell nuclei, which could be attributed to the stronger cell traction force and softer nuclear stiffness. These two biomechanical changes were correlated, respectively, to a larger amount of focal adhesion and less amount of nuclear lamins. Taken together, our works revealed not only the biomechanical profiles of LN-metastatic cells but also the corresponding biomolecular expressions to pinpoint the key process in LN metastasis.

## Introduction

Lymph node (LN) metastasis is the major risk and cause of deaths in patients with head and neck squamous cell carcinoma (HNSCC) (Noguti et al., 2012). Up to 50% of HNSCC patients have LN metastasis in which cancer cells detach from primary tumor tissues (oral, tongue, or throat site), invade into surrounding extracellular matrices (ECMs), spread to lymph nodes, damage the lymph nodes, and further metastasize to regional nodes (Shah et al., 1990). Accumulated evidence reveals that LN metastasis has high risk for poor prognosis and survival of HNSCC patients (Cerezo et al., 1992; Rudoltz et al., 1995; Ho et al., 2017). Hence, the questions of “how HNSCC cells manage to invade lymph nodes from the primary tumor site?” and “what are the differences between LN-metastatic and non-metastatic HNSCC cells in their biochemistry and biomechanics?” deserve further investigations.

Tumor-induced lymphangiogenesis is crucial for tumor growth and LN metastasis (Hirakawa et al., 2007). The vascular endothelial growth factor-C (VEGF-C) secreted from malignant tumor cells is the major regulator in tumor-induced lymphangiogenesis to promote LN metastasis (Hirakawa et al., 2007; Kudo et al., 2012; Chen et al., 2013). The VEGF-C enhances the formation of tumor lymphatic vessels surrounding tumors and activates the integrin α4β1 on the endothelium of the tumor lymphatic vessels which interacts with vascular cell adhesion molecule 1 (VCAM-1) to promote cancer cell migration and LN metastasis (Garmy-Susini et al., 2013). Snail, a transcription factor, plays a critical role in enhancing tumor progression through epithelial–mesenchymal transition (EMT), as well as in the generation and collective movement of circulating tumor cell (CTC) clusters. In addition, Snail also enhances angiogenesis by upregulating VEGF expression (Shin et al., 2012; Chang et al., 2020). Hence, in HNSCC, Snail has been recognized as an important biomarker for LN metastasis (Ginos et al., 2004; Mendelsohn et al., 2012). To date, previous studies have focused primarily on various snail-regulated signal transduction and signaling molecules during LN metastasis (Olmeda et al., 2007; Shin et al., 2012). However, the change in the biomechanical properties of HNSCC cells during LN metastasis remains elusive, and how Snail regulates the corresponding mechanical properties of cancer cells to promote LN metastasis has attracted much less attention.

**FIGURE 1:**
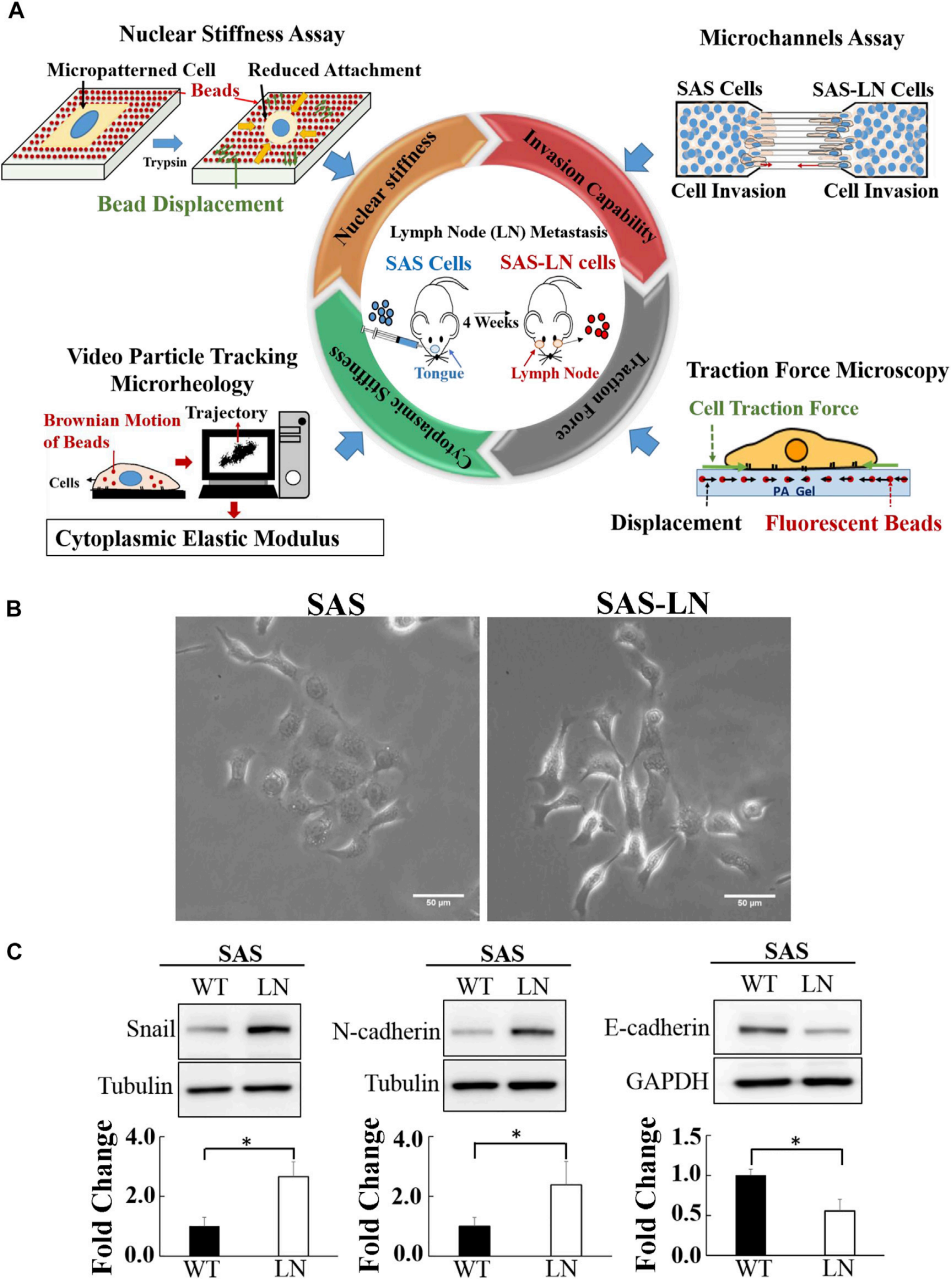
Characterization of the biomechanical properties and the expression of epithelial–mesenchymal transition marker proteins of lymph node (LN)-metastatic and non-metastatic head and neck squamous cell carcinoma (HNSCC) cells (SAS-LN and SAS cells). **(A)** A schematic diagram of multiple biophotonics platforms to quantify the biomechanical properties of SAS-LN and SAS cells, including nuclear stiffness via nuclear stiffness assay; invasion capability via a microchannel assay with different channel widths (12, 16, and 20 μm); cytoplasmic stiffness via video particle tracking microrheology; and cell traction force via traction force microscopy. **(B)** Phase contrast images of LN-metastatic and non-metastatic HNSCC cells (SAS-LN and SAS cells). Scale bar = 50 μm. **(C)** Western blot of Snail, N-cadherin, and E-cadherin in SAS-LN and SAS cells and fold change expression relative to SAS cells. Tubulin and GAPDH were used as loading control. Data represent mean ± SD (n = 3). *, for *p* < 0.05 and **, for *p* < 0.01.

In physiology and pathology, the alteration of the mechanical properties of cancer cells, including cytoplasm stiffness, cell traction force, and nuclear stiffness, plays a crucial role in cancer invasion and metastasis (Wirtz, Konstantopoulos, and Searson, 2011; Lintz et al., 2017; Kim et al., 2018). In general, tumor tissues are stiffer than normal healthy tissue, such as those in breast, colorectal, and head and neck cancer, which is associated with an increase in ECM stiffness through increasing collagen deposition and crosslinking within the tumor stroma (Carey et al., 2012; Wolf et al., 2013; Peltanova et al., 2019). Our previous studies showed that a high-concentration collagen matrix exhibited a compact and stiff structure with small pore size, which limited force-induced collagen remodeling and invasion of head and neck cancer cells (OECM-1) (Chen et al., 2020). The capability of cancer cells to successfully invade into the smaller pore size of tumor microenvironments, including the surrounding ECM, blood vessels, and lymph nodes, relies critically on their biomechanical properties (Wirtz et al., 2011; Emon et al., 2018). Therefore, we aimed to investigate the biomechanical and biomolecular properties of LN-metastatic and non-metastatic HNSCC cells. In this study, we used a lymph node-metastatic mouse model of human HNSCC to generate SAS-LN cells to mimic the lymph node metastasis of SAS cells. To investigate the biomechanical properties of SAS cells (non-metastatic HNSCC cells) and SAS-LN cells (LN-metastatic HNSCC cells), we measured their 1) cellular traction force via traction force microscopy (TFM) (Lee et al., 2013; Mark et al., 2020); 2) cytoplasmic stiffness via video particle tracking microrheology (VPTM) (Wirtz, 2009; Chen et al., 2018); 3) cellular nuclear stiffness via nuclear stiffness assay (Versaevel et al., 2012); and 4) invasion capability via microchannels assay (Riahi et al., 2014; Holle et al., 2019), as is illustrated in Figure 1A and Supplementary Figure S1. We further examined the expression of Snail in SAS-LN and SAS cells and found that Snail expression was higher in SAS-LN (LN-metastatic cells), supporting the notion that Snail is crucial for the alterations of biomolecular and the corresponding biomechanical properties of cancer cells for LN metastasis. In addition, we found that the overexpression of Snail in SAS cells caused stronger cell traction force and more compliable cell nuclei, resulting in an enhanced longitudinal strain of cell nuclei, to promote cancer invasion in narrow microchannels. These Snail-induced biomechanical changes could be attributed to the elevated number of large focal adhesion-associated protein and suppressed expression of nuclear lamins at the nuclear periphery. Taken together, these findings provide a more comprehensive view of the biomechanical and biomolecular mechanisms, correlated with Snail expression, to better understand the progression of LN metastasis of HNSCC.

## Materials and Methods

### Cell Lines and Plasmids

The human head and neck squamous cell carcinoma (HNSCC) cell lines (OEC-M1 and SAS) were provided by Professor Muh-Hwa Yang, Institute of Clinical Medicine, National Yang Ming Chiao Tung University. OEC-M1 and SAS cells were maintained in the medium of RPMI 1640 (11875-093, Gibco) and DMEM (11995-065, Gibco), respectively, supplemented with 10% FBS (10270106, Gibco) and 1% penicillin–streptomycin solution (15140-122, Gibco) at 37°C and 5% CO_2_. The SAS-Snail cell line was generated by transfecting PCDH-puro-Snail plasmid and PCDH-puro-ctrl; the latter served as a control (Li et al., 2019). For Snail silencing, pLKO-shCtrl and pLKO-shSnail, obtained from the National RNAi Core Facility of Taiwan, were used in SAS-LN (Li et al., 2019).

### *In Vivo* Generation of the Lymph Node Sub-Cell Line

Lymph node (LN)-metastatic HNSCC cells (SAS-LN) were generated and provided by Professor Muh-Hwa Yang (Institute of Clinical Medicine, National Yang Ming Chiao Tung University) (Li et al., 2019). In brief, LN-metastatic HNSCC cells (SAS-LN), isolated from the lymph nodes of nude mice, 4 weeks after SAS cells were injected into their tongue, were maintained in DMEM supplemented with 10% FBS and 1% penicillin–streptomycin solution at 37°C and 5% CO_2_. Cell samples for all the experiments were within 10 passages. The expressions of Snail, N-cadherin, and E-cadherin in passages 1 and 10 did not have significant differences (Supplementary Figure S2).

### Western Blot Analysis

Cells were lysed in the protease inhibitor-containing lysis buffer for western blot analysis. The protein lysates were separated by SDS-PAGE, transferred onto a PVDF membrane (Millipore Corp., Bedford), and stained with proper antibodies. The antibodies used in this study include the antibodies against Snail (3895, Cell Signaling), E-cadherin (3195, Cell Signaling), N-cadherin (610920, BD), lamin A/C (8984, Abcam), lamin B1 (16048, Abcam), tubulin (T6074, Sigma-Aldrich), and GAPDH (2118s, Cell Signaling). Signals were developed using an enhanced chemiluminescence kit (Millipore Corp.) and photographed using Fujifilm LAS-4000.

### Microchannels

The invasion capability of SAS-LN and SAS cells was assessed via microchannels (MC005, 4DCELL) of 5 μm height and varying widths (12, 16, and 20 μm). In total, 15 μL of cell suspension (10^7^ cell/mL) was added into the access ports of the microchannels and incubated at 37°C for 24 h. The invasion capability was determined by counting the number of cells invading into the microchannels from the access ports. In our microchannel assay, SAS-LN and SAS cells migrated into the microchannels from their opposite ends. We have demonstrated that the presence of each type of cell on the opposite sites of the microchannels did not affect cell invasion, which implied the absence of a significant interaction between SAS and SAS-LN cells on cell invasion in our microchannel assay (Supplementary Figure S3). A microchannel assay, with different channel widths, is a robust method to analyze the directional invasion of cells *in vitro*. However, physiologically, cell invasion involves the cell–ECM interaction, including force remodeling of the ECM, and MMP-mediated ECM degradation; hence, the microchannel assay lacks the information associated with cell–ECM interaction.

### Micropattern-Based Nuclear Stiffness Assay

The micropattern-based nuclear stiffness assay, consisting of traction force microscopy and rectangular micropattern to simultaneously measure the cell traction force and the longitudinal strain of cell nucleus and then calculate the nuclear stiffness, was described in a previous report (Versaevel et al, 2012). In brief, the rectangular micropattern on top of polyacrylamide gels was fabricated as follows. Polyacrylamide gel was prepared by mixing 0.1% of Bis (1610142, Bio-Rad) with 10% of acrylamide (1610140, Bio-Rad) to obtain 9 kPa gels and mixed with 200 nm-diameter fluorescent beads. Rectangular micropattern arrays (length = 55 μm and width = 18 μm) were fabricated on a silicon wafer by using deep reactive-ion etching. PDMS gel was poured onto the silicon wafer and cured at 80°C for 1h to form PDMS stamps. PDMS stamps were coated with 50 μg/ml of fibronectin and then pressed onto a polyacrylamide-coated glass coverslip to fabricate fibronectin-coated adhesive islands on top of the polyacrylamide gel. Cells were cultivated on the nuclear stiffness assay of rectangular micropattern at 37°C for 4 h to allow for cell adherence (Supplementary Figure S4). Fluorescent images of the cell nuclei and fluorescence beads of cells, with vs. without trypsin treatment, seated on the surface of the rectangular micropattern, were recorded using an epi-fluorescence microscope system (Eclipse Ti, Nikon) with a 40× oil-immersion objective (MRH01401, Nikon; numerical aperture = 1.3). The displacements of particles were analyzed by PIVlab software (Thielicke and Stamhuis, 2014). The algorithm of TFMatlab software was used to calculate traction forces (Beningo and Wang, 2002; Kovari et al., 2016). The 200 nm-diameter fluorescent beads, embedded in polyacrylamide substrates, serve as the probes for the detection of substrate deformation in response to cell traction forces. As cells exerted forces on the substrates and induced the substrate deformation, the embedded beads were displaced accordingly. Thus, the analysis of bead displacements could be used to measure the substrate deformations and further deduce the cell traction forces. The nuclear stiffness was simply deduced (by Hooke’s Law) from the longitudinal strain of cell nucleus and cellular traction force along the major axis of the rectangular micropattern. Traction force microscopy (TFM) is a useful method to measure the spatial distribution of cell traction forces, for cells seeded on a 2D polyacrylamide gel. However, TFM has a limitation for the quantification of cell traction forces in 3D ECM environments.In addition, the TFM method is applicable only to homogeneous and linear elastic material with a linear strain–stress response (Plotnikov et al., 2014). When cells are cultured in a 3D ECM, such as 3D collagen matrices, cells could secrete enzymes to degrade the collagen fibers, to bear non-uniform mechanical properties. Hence, the nuclear stiffness assay, consisting of TFM and micropattern, is applicable for the quantification of the nuclear stiffness of cells, only in 2D environments, but not in a 3D ECM.

### Video Particle Tracking Microrheology

Video particle tracking microrheology (VPTM) was used to analyze the cytoplasm stiffness as was described in our previous reports (Chen et al., 2016; Chen et al., 2018; Yu et al., 2015). We injected red fluorescent nanoparticles (F8807, Invitrogen, fluorescence excitation/admission peaks: 580 nm/605 nm, diameter = 200 nm, concentration: 1.35×10^12^ particles/mL) into the cells via a biolistic particle delivery system (PDS-100, Bio-Rad; pressure 450 psi). The Brownian motion of the beads was tracked and recorded via an epi-fluorescence microscope system (Eclipse Ti, Nikon), equipped with a ×100 oil-immersion objective (MRD01991, Nikon, numerical aperture = 1.45) and a CMOS camera (Hamamatsu, OHCA-Flash 4.0; 100fps). The mean squared displacement (MSD) <Δr ^2^(τ)> = <[x (t + τ) − x(t)]^2^ + [y (t + τ) − y(t)]^2^ > was calculated from the two-dimensional trajectory, x(t) and y(t), of each bead. The effective creep compliance J(τ) and the elastic modulus G’ (*ω*) were deduced from MSD (Baker et al., 2009), as shown in Eqs. 1, 2, where “*a*” is the particle’s radius, K_B_ the Boltzmann constant, and T is the absolute temperature. The cytoplasmic stiffness was compared in terms of the value of the elastic modulus G′(*ω*) at frequency f = ω/2π = 10 Hz.

$$\;J\left(\text{τ}\right)=\frac{\pi a}{K_{B}T}<\Delta r^{2}\left(\tau \right)>,$$ (1)

$$G^{\prime }\left(\omega \right)=\left[J{\left(\text{τ}\right)}^{-1}\right].$$ (2)

VPTM measures the local elastic modulus of cells based on the Brownian motion of individual intracellular particles without external contact forces. Hence, it has a great advantage in measuring the elastic modulus of cells cultured in 3D ECM environments. However, without applied external forces, it is a challenge to use VPTM to measure viscoelastic properties of materials in a non-equilibrium condition (Wirtz, 2009).

### Immunofluorescence Staining

The primary antibodies used in this experiment are 1:200 dilution of anti-lamin A/C mouse monoclonal antibody (ab8984, Abcam), 1:1000 dilution of anti-lamin B1 rabbit polyclonal antibody (ab16048, Abcam), 1:200 dilution of anti-SNAI1 (GTX100754, GeneTex), and 1:200 dilution of anti-paxillin rabbit polyclonal antibody (GTX125891, GeneTex). The cell nuclei and F-actin were stained with 5 μg/mLof Hoechst 33342 (H3570, Thermo Fisher Scientific) and 165 nM of rhodamine–phalloidin (R415, Thermo Fisher Scientific), respectively. Immunofluorescent images were taken using a laser scanning microscope system (LSM 880, ZEISS) equipped with a 40× oil-immersion objective (plan-apochromat, ZEISS; numerical aperture = 1.3).

### Image Analysis

The individual focal adhesion (FA) area and number of FAs per cells were analyzed by MetaMorph software. The nuclear morphology was characterized in terms of nuclear area and nuclear aspect ratio (the ratio of the length of major and minor axes) via ImageJ software. The number of cells in the microchannel was analyzed by ImageJ software. The co-localization of paxillin and actin filaments was analyzed using ImageJ software.

### Statistical Analysis

Statistical analysis of data was performed using MATLAB and carried out by one-way ANOVA, followed by Tukey’s test. The statistical significance at *for *p* < 0.05 and **for *p* < 0.01 and N.S. (not significant) are shown in the figures.

## Results

### LN-Metastatic HNSCC Cells Elevated Snail Expression and Exhibited More Mesenchymal Phenotype

In this study, a lymph node metastatic mouse model system was used to generate LN-metastatic human HNSCC cells (SAS-LN), which were isolated from lymph nodes of nude mice in 4 weeks after tongue injection with non-metastatic HNSCC cells (SAS). In contrast to SAS cells, SAS-LN cells displayed a more mesenchymal phenotype with elongated morphology (Figure 1B) and exhibited higher levels of Snail expression (Figure 1C and Supplementary Figure S5A, B) and mesenchymal marker N-cadherin and a lower level of the epithelial marker E-cadherin (Figure 1C). These results suggested that Snail expression was associated with LN metastasis of HNSCC tumor.

### LN-Metastatic Cells Demand Distinct Biomechanical Properties

**FIGURE 2:**
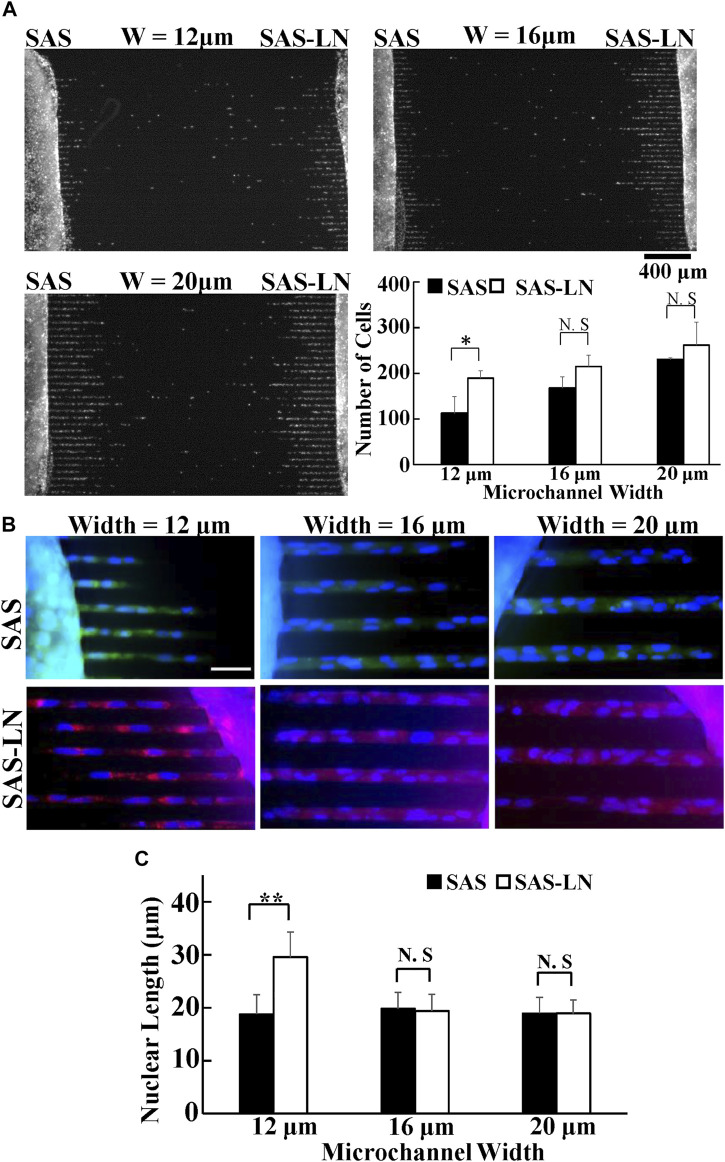
Invasion capability of LN-metastatic HNSCC cells (SAS-LN) and non-metastatic HNSCC cells (SAS). **(A)** The numbers of SAS-LN and SAS cells, after invasion for 24 h from the access ports into the microchannels (with different widths). To visualize and count the number of cells in the microchannels, the cell nuclei were stained with Hoechst 33342. Data represent mean ± SD (n = 3). Scale bars = 400 μm. **(B)** The nuclear morphology of cells invasion into 12 μm, 16 μm, and 20 μm channels. SAS-LN cells were stained with celltracker Red CMTPX Dye (shown in red), SAS cells were stained with celltracker CMFDA Dye (shown in green), and cell nuclei were stained with Hoechst 33342 (shown in blue). Scale bars = 50 μm. **(C)** The nuclear length of SAS-LN and SAS cells, in 12, 16, and 20 μm channels. Data represent mean ± SD (n = 50). *, for *p* < 0.05 and **, for *p* < 0.01; N.S: not significant.

To comprehensively and systematically investigate the difference in the biomechanical properties of SAS-LN and SAS cells, we used microchannels, traction force microscopy (TFM), video particle tracking microrheology (VPTM), and nuclear stiffness assay to measure 1) cells’ invasion capability, 2) cells’ traction force, 3) cytoplasmic stiffness, and 4) nuclear stiffness (Figure 1A), respectively. The invasion capability was examined via microchannels with different channel widths (12, 16, and 20 μm) (Figure 2A). We found that SAS-LN cells exhibited higher invasion capability in narrow microchannels (width = 12 μm) compared with SAS cells; the number of SAS-LN cells invading into narrow microchannels (width = 12 μm) was much higher than that of SAS cells. In contrast, for both types of cells, the number of cells invading through the wider microchannels (16 and 20 μm) was comparable (Figure 2A); hardly any cells invaded into microchannel widths below 8 μm (Supplementary Figure S6). Meanwhile, we also observed that compared to the nuclei of SAS cells, the nuclei of SAS-LN cells were more elongated (quantified in term of the aspect ratio; i.e., major axis length/minor axis length of the cell nucleus) in narrow microchannels (width = 12 μm) (Figures 2B,C). These results implied that the higher invasion capability of LN-metastatic HNSCC cells (i.e., SAS-LN cells) in narrow microchannels could be correlated with their more elongated morphology. We further determined the area, volume, and aspect ratio of the cell nuclei in SAS and SAS-LN and found that the values of these parameters are comparable when cells were cultured on a glass-bottom dish (Supplementary Figure S7), indicating that the higher invasion capability of SAS-LN cells was not caused by the nuclear size and nuclear morphology.

**FIGURE 3:**
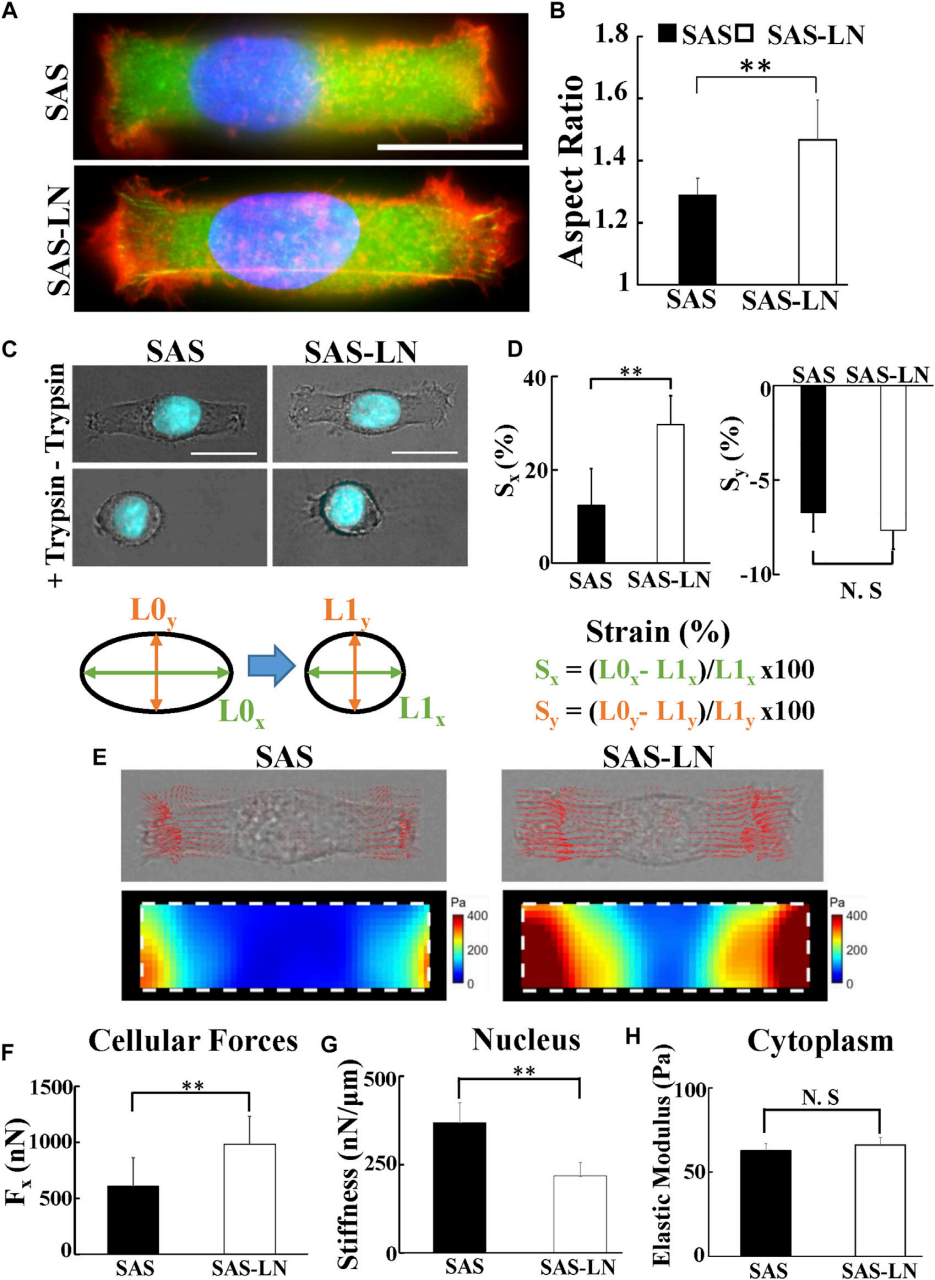
The biomechanical properties of LN-metastatic HNSCC cells (SAS-LN) and non-metastatic HNSCC cells (SAS). **(A)** Representative immunofluorescence images of SAS-LN and SAS cells cultured on the rectangular micropatterns. Blue: nucleus (Hoechst 33342), red: TRITC-labeled phalloidin for actin, and green: FITC-labeled paxillin. **(B)** Analysis of the nuclear morphology of SAS-LN and SAS cells, cultured on the rectangular micropatterns (length = 55 μm and width = 18 μm). Data represent mean ± SD (n = 20). *, for *p* < 0.05 and **, for *p* < 0.01; N.S: not significant. **(C)** Phase contrast and fluorescent images of nuclear morphology of SAS-LN cells and SAS cells, cultured on the rectangular micropatterns, before (upper panels) and after (lower panels) trypsin treatment. Blue: cell nucleus. Scale bars = 20 μm. **(D)** Analysis of the longitudinal (S_x_) and transverse (S_y_) strain of nuclei, along the major axis and the minor axis of the rectangular micropatterns, respectively, in SAS-LN cells and SAS cells before and after trypsin treatment. **(E)** Representative images showing 2D traction stress vectors (upper panels) and the corresponding magnitude distributions (lower panels) of SAS-LN cells and SAS cells. Traction stress, T, was calculated from the displacement of fluorescence beads before and after trypsin treatment to reduce cell adhesion. White dotted boxes show the periphery of the rectangular micropatterns. **(F)** Quantitative comparison of total traction force generated by SAS-LN cells and SAS cells. Traction forces were calculated by integrating magnitudes of traction stress (T) over the projected cell area. Elongation forces (F_x_) repesent the reaction of traction forces projected along the major axis of the rectangular micropatterns. **(G)** Quantitative comparison of the nuclear stiffness of SAS-LN cells and SAS cells. The nuclear stiffness was deduced from the longitudinal strain of cell nucleus along the major axis of rectangular micropatterns and the elongation forces (F_x_) according to Hooke’s law. Data represent mean ± SD (n = 20). **(H)** The cytoplasmic stiffness (at frequency f = 10 Hz) of SAS-LN cells and SAS cells, measured by VPTM. Data represent mean ± SD (n = 20).

Previous studies have indicated that the cellular strain is determined by its unique stiffness, including nuclear stiffness and cytoplasmic stiffness (McGregor et al., 2016). Here, we used nuclear stiffness assay and video particle tracking microrheology (VPTM) to quantify the nuclear stiffness and cytoplasmic stiffness, respectively, of SAS-LN and SAS cells. The nuclear stiffness assay consisted of rectangular micropattern and traction force microscopy. The elongation of cell nuclei, in response to seeding on a rectangular micropattern (length = 55 μm and width = 18 μm), was measured by epi-fluorescence microscopy and subsequent image analysis; the elongation force (i.e., reaction of the traction force on the cell by the substrate) was measured by traction force microscopy; the cell nuclear stiffness was calculated from the nuclear elongation length and the elongation force. Using this approach, we observed that the nuclear morphology of SAS-LN cells, in comparison with that of SAS, exhibited larger elongation along the major axis of the rectangular micropattern (Figures 3A,B) and high expression of Snail (Supplementary Figures S5C–E); both types of cells showed a positive correlation of nuclear elongation with Snail expression (Supplementary Figure S5F). In addition, the nuclei of SAS-LN cells were thinner and had smaller volume, compared with those of SAS cells (Supplementary Figure S8A, B). When cells were treated with 0.1% of trypsin for 5 min to reduce cell adhesion, the cell nuclei became rounder (Figure 3C). The strain of cell nuclei, deduced from the length difference of the cell nuclei before and after trypsin treatment, also showed that the longitudinal strain of the cell nuclei in SAS-LN cells was higher than that of SAS cells (Figure 3D). The high longitudinal strain of cell nuclei could be attributed to either stronger elongation forces, or more pliable cell nuclei, or their combination. Hence, we further quantified elongation forces (i.e., reaction of traction forces projected along the major axis of the rectangular micropattern) and calculated the nuclear stiffness of SAS-LN and SAS cells. We observed that SAS-LN cells exerted stronger force at the edges of the elongated cells (Figures 3E,F), and the cell nuclei were more pliable compared with SAS cells (Figure 3G). In contrast, there was no significant difference in the cytoplasmic stiffness between SAS-LN and SAS cells (Figure 3H).

Actin cytoskeleton is one of the major cytoskeletal components that mediates cell shape and cell motility and participates in the regulation of cell traction forces and nuclear movement and deformation (Hui et al., 2015; Svitkina, 2018; Fischer et al., 2020). To examine how actin polymerization affects the biomechanical properties of the LN-metastatic (SAS-LN) vs. those of the non-metastatic (SAS) cells, we measured the traction forces and the nuclear deformation of SAS and SAS-LN cells, with and without the treatment of 0.2 μM latrunculin-A (Lat-A) for 1 h to inhibit actin polymerization. We observed that the inhibition of actin polymerization induced the collapse of the cell membrane, the reduction of nuclear elongation, and cell elongation forces; furthermore, the reduction of nuclear deformation and cell elongation forces were more pronounced in SAS-LN cells than those in SAS cells (Supplementary Figure S9). These results indicated that, in comparison with SAS cells, strain of the nuclei in SAS-LN cells relied more on the transmission of contractile force from actin filaments.

### Change in Biomolecular Properties Is Concomitant With Change in Biomechanical Properties in LN Metastasis

**FIGURE 4:**
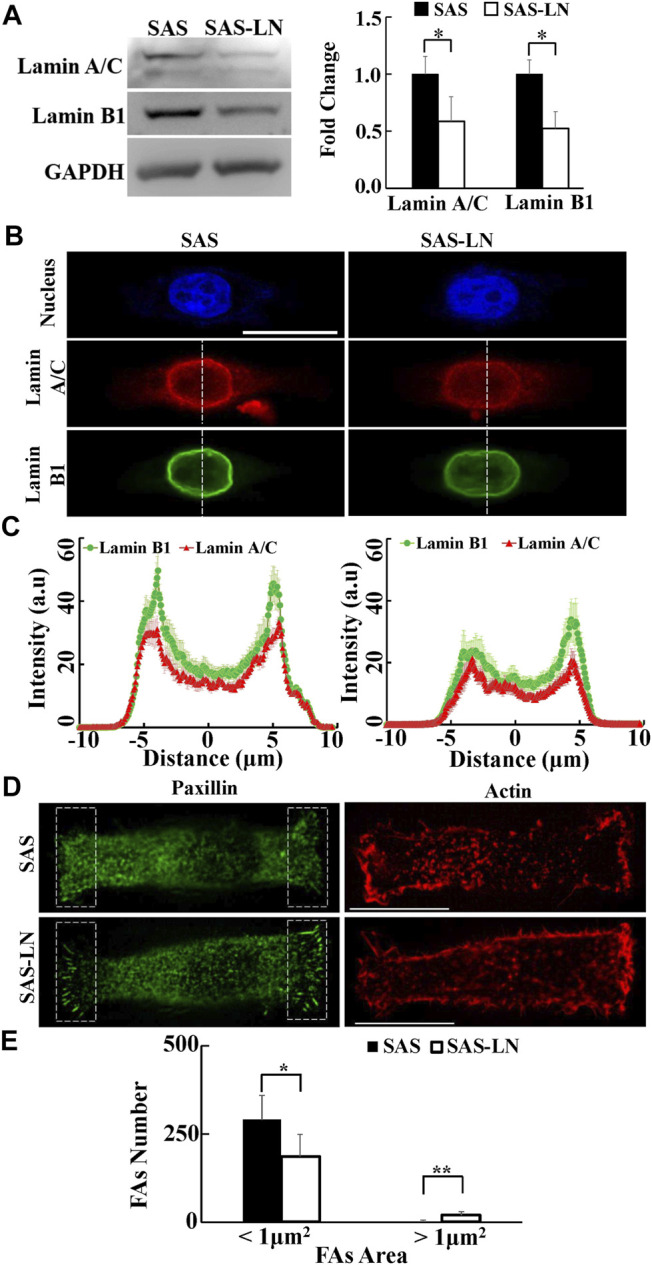
Characterization of the nuclear biomolecules and focal adhesion size of LN-metastatic HNSCC cells (SAS-LN) and non-metastatic HNSCC cells (SAS). **(A)** Left: western blot of lamin A/C and B1 in SAS-LN and SAS cells. GAPDH was used as a loading control. Right: fold change expression of lamin A/C and B1 relative to SAS cells. Data represent mean ± SD (n = 3). *, for *p* < 0.05 and **, for *p* < 0.01. **(B)** Representative immunofluorescence images of the cell nuclei, lamin A/C and B1 in SAS-LN and SAS cells. Scale bars = 20 μm. **(C)** Quantitative measurement of the distribution profiles of lamin A/C and B1, indicated by the fluorescence intensity distributions, in SAS-LN and SAS cells, along the dotted lines across the cell nuclei as shown in (B). Data represent mean ± SD (n = 20). **(D)** Immunofluorescence micrographs of focal adhesion proteins paxillin and actin filaments in SAS-LN and SAS cells. Scale bars = 20 μm. **(E)** A comparison of the numbers of focal adhesions (FAs), with FAs area >1 μm^2^ vs. those with FAs area <1 μm^2^, in SAS-LN and SAS cells, in the regions (8 μm × 20 μm) bounded by the white dotted rectangles shown in **(D)**. All data are expressed as mean ± SD from 15 cells.

Previous studies have indicated that the biomechanical properties of cells are regulated by the corresponding biomolecular expression, including lamin, focal adhesions (FAs), and actin cytoskeleton (Gardel et al., 2010). Nuclear structure and stiffness are maintained mainly by lamins (Alisafaei et al., 2019); cell traction force is associated with focal adhesion maturation, actin filament organization, and myosin II activation (Gardel et al., 2010); and the number of mature FAs, with size >1 μm^2^, has a positive correlation with the magnitude of local traction force (Balaban et al., 2001; Goffin et al., 2006). In addition, the cytoskeleton organization plays an important role in maintaining the cell morphology and cytoplasmic stiffness (Schakenraad et al., 2020). To further investigate the linkage between the biomechanical properties of LN-metastatic cells and LN metastasis, we analyzed the expressions and distributions of paxillin, lamin A/C and B1, and actin filaments of SAS-LN and SAS cells. Our results showed that SAS-LN cells displayed lower expressions of lamin A/C and lamin B1, compared with SAS cells (Figure 4A). We further quantified the distribution of fluorescence intensities of lamin A/C and lamin B1 at the nuclear periphery and nuclear center. The fluorescence intensities of lamin A/C and lamin B1 at the nuclear periphery in SAS-LN cells were significantly lower than those in SAS cells (Figures 4B,C). Next, SAS and SAS-LN cells were stained with paxillin to visualize the FAs. In comparison with SAS cells, the SAS-LN cell displayed a larger number of paxillin-marked FAs (paxillin area >1 μm^2^), a smaller number of paxillin-marked FAs (paxillin area < 1 μm^2^) (Figures 4A,D,E), and higher degree of co-localization of paxillin and actin filaments at the cell edges (Supplementary Figure S10A–D). In contrast, the distributions of actin filaments in SAS-LN cells and in SAS cells were similar; both displayed fewer actin stress fibers with significant cortical actin filaments at the cell periphery (Figure 4D). These experimental results supported our speculation that the LN metastasis was associated with stronger traction force and more compliable nuclei of LN-metastatic cells (SAS-LN), concomitant with a larger number of FAs and suppressed expressions of nuclear lamin A/C and B1.

### Snail Regulates the Biomechanical Properties of HNSCC Cells for LN Metastasis

**FIGURE 5:**
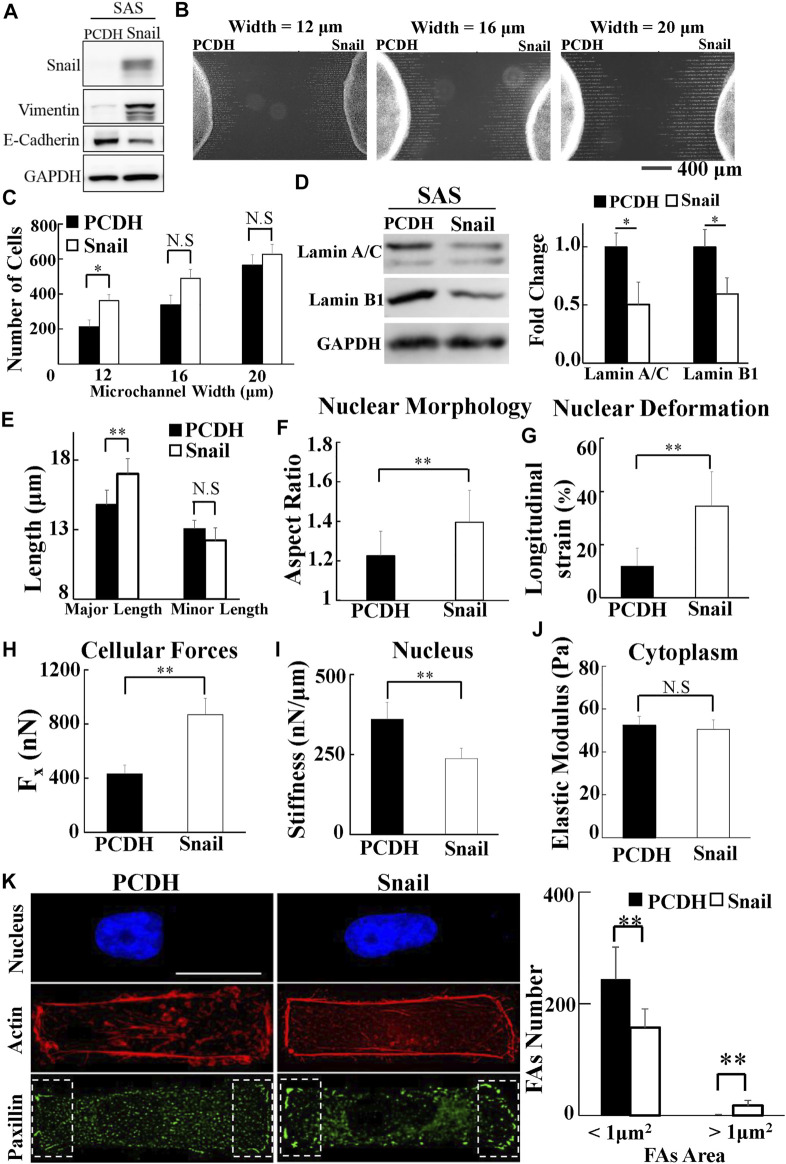
The effect of Snail on the biomolecular and biomechanical properties of SAS cells. **(A)** Western blot of Snail, E-cadherin, and vimentin in control (PCDH) by transfecting PCDH-puro-ctrl and Snail-overexpressing SAS cells (SAS-Snail) by transfecting PCDH-puro-Snail plasmid, with GAPDH as a loading control. **(B)** The fluorescence images of SAS-Snail and SAS cells, after invasion for 24 h from the access ports into the microchannels (with different widths of 12, 16, and 20 μm). To visualize and count the number of cells in the microchannels, the cells nuclei were stained with Hoechst 33342. **(C)** Overexpression of Snail enhanced the invasion capability of SAS cells in a narrow channel (12 μm). Data represent mean ± SD (n = 3). **(D)** Overexpression of Snail suppressed the expression of lamin A/C and B1. Left: western blot of lamin A/C and B1 in control (PCDH) and Snail-overexpressing SAS cells (SAS-Snail). GAPDH was used as a loading control. Right: fold change of lamin A/C and B1 expressions relative to control (PCDH) cells. Data represent mean ± SD (n = 3). *, for *p* < 0.05 and **, for *p* < 0.01. **(E–J)** Overexpression of Snail elongated the nuclear morphology in response to the rectangular micropattern, enhanced the longitudinal strain of cell nuclei and the elongation forces (F_x_), and reduced the cell nuclear stiffness, with much smaller effect in cytoplasmic stiffness. Data represent mean ± SD (n = 15). **(K)** Overexpression of Snail raised the number of mature FAs (FAs area >1 μm^2^) and reduced the number of nascent FAs (FAs area <1 μm^2^); however, its effect on actin filaments was much less. Left: immunofluorescence micrographs of nuclear actin and paxillin filaments of control (PCDH) and Snail-overexpressing SAS cells (SAS-Snail). Scale bars = 20 μm. Right: the numbers of focal adhesions (FAs) in the regions (8 μm × 20 μm) bounded by the white dotted rectangles shown in the lower panel in **(K)**. All data are expressed as mean ± SD from 15 cells.

To investigate whether Snail could regulate the biomolecular and biomechanical properties of HNSCC cells for LN metastasis, we examined the effect of Snail overexpression on the biomolecular (Figure 5A) and the corresponding biomechanical properties of SAS cells. We systematically compared the invasion capability, cell traction force, cytoplasmic stiffness, and nuclear stiffness of SAS cells vs. Snail-overexpressed SAS cells. Our results showed the upregulated Snail expression enhanced invasion capability in a narrow microchannel (12 μm) (Figures 5B,C). A smaller amount of lamin A/C and B1 was observed in Snail-overexpressed SAS cells compared with SAS cells (Figure 5D). In addition, the overexpression of Snail also reduced the nuclear stiffness, thereby enhancing the nuclear elongation and longitudinal strain in response to cell culture on a rectangular micropattern (Figures 5E–I). Although there was no significant difference in cytoplasm stiffness and actin distribution (Figures 5J,K), we found that the Snail expression upregulated cell traction force, resulting in a larger number of large paxillin-marked FAs (paxillin area >1 μm^2^) and a smaller number of small paxillin-marked FAs (paxillin area <1 μm^2^) (Figure 5K) and a higher degree of co-localization of paxillin and actin filaments at the cell edges (Supplementary Figure S10E). These results indicated that Snail expression was responsible for the transmission of contractile force from actin filaments to substrates through mature FAs. Stronger cell traction force and more deformable cell nuclei supported the elevated invasion capability of Snail-overexpressed SAS cells.

**FIGURE 6:**
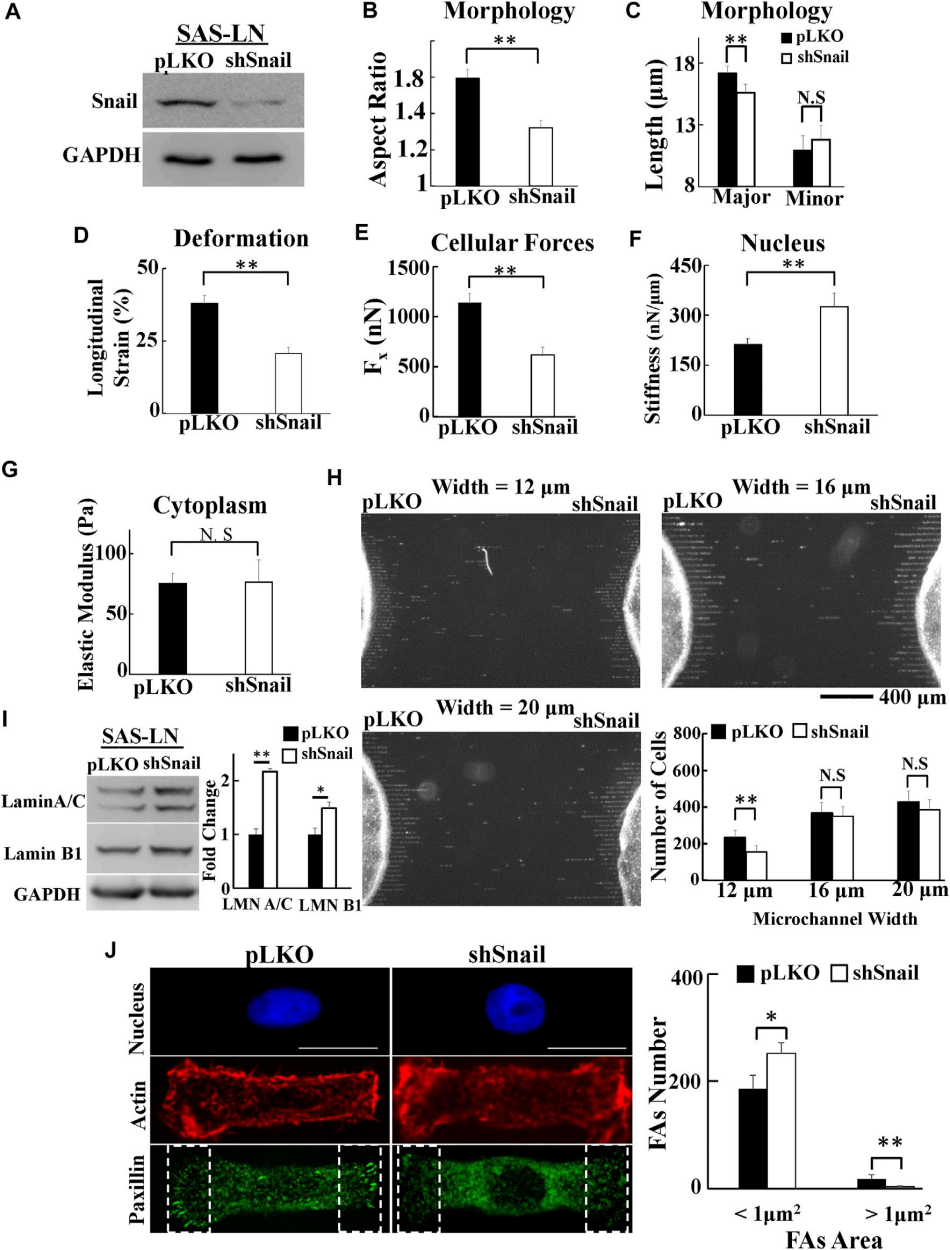
The effect of Snail-knockdown on the biomolecular and biomechanical properties of LN-metastatic HNSCC cells (SAS-LN). **(A)** Western blot of Snail in control (SAS-LN-pLKO) using pLKO-shCtrl and Snail1-knockdown SAS-LN cells (SAS-LN-shSnail) using pLKO-shSnail, with GAPDH as a loading control. **(B-C)** Knockdown of Snail induced a round morphology of cell nuclei. **(D-G)** Knockdown of Snail reduced the longitudinal strain of cell nuclei and elongation forces (F_x_), enhanced nuclear stiffness, but rendered smaller effect on cytoplasmic stiffness. Data represent mean ± SD (n = 15). **(H)** The numbers of SAS-LN-shSnail and SAS-LN-pLKO cells, after invasion for 24hrs from the access ports into the microchannels (with different widths of 12, 16, and 20 μm). To visualize and count the number of cells in the microchannels, the cell nuclei were stained with Hoechst 33342. The lower right panel shows the invasion capability of SAS-LN-shSnail and SAS-LN-shluc cells in the microchannels. Data represent mean ± SD (n = 3). **(I)** Knockdown of Snail enhanced the expression of lamin A/C and B1. Left: western blot of lamin A/C and B1 in control (pLKO) and Snail1-knockdown SAS-LN cells (SAS-LN-shSnail). GAPDH was used as a loading control. Right: fold change expression of lamin A/C and B1 relative to the control (pLKO) cells. Data represent mean ± SD (n = 3). *, for *p* < 0.05 and **, for *p* < 0.01. **(J)** Knockdown of Snail reduced the number of mature FAs (FAs area >1 μm^2^) and augmented the nascent FAs (FAs area <1 μm^2^). Left: immunofluorescence micrographs of the cell nuclei, paxillin, and actin filaments of control (SAS-LN-pLKO) and Snail-knockdown SAS-LN cells (SAS-LN-shSnail). Scale bars = 20 μm. Right: the numbers of focal adhesions (FAs) in the regions (8 μm × 20 μm) bounded by the white dotted rectangles in the lower panel in **(J)**. All data are expressed as mean ± SD from 15 cells.

To further confirm that Snail can contribute to increase cell traction force and soften nuclear stiffness, we used shRNA sequences to inhibit Snail in LN-metastatic HNSCC cells, SAS-LN (Figure 6A). Consistently, we found that knockdown of Snail reduced the nuclear elongation (Figures 6B,C), longitudinal strain of cell nuclei (Figure 6D), and cell traction force (Figure 6E). In addition, it boosted the stiffness of the cell nuclei (Figure 6F) but did not affect the cytoplasmic stiffness (Figure 6G). We also found that knockdown of Snail suppressed the cell invasion capability in narrow microchannels (12 μm) (Figure 6H). In comparison with SAS-LN cells, more lamin A/C and B1 (Figure 6I), a smaller number of large paxillin complexes (paxillin area >1 μm^2^), a larger number of small paxillin complexes (paxillin area <1 μm^2^) (Figure 6J), and a lower degree of co-localization of paxillin and actin filaments at the cell edges were observed in Snail-knockdown SAS-LN cells (Supplementary Figure S10F).

Consistently, Snail knockdown produced similar effects in another mesenchymal HNSCC cell line, OEC-M1 (Supplementary Figure S11). These experimental results revealed how Snail regulated the biochemical and biomechanical properties of HNSCC cells to promote their LN invasion capability.

## Discussion

Lymph node (LN) metastasis is a complex process which requires coordinated changes in the biomolecular and biomechanical properties of cancer cells to invade from primary tumor to the surrounding ECM, tissues, blood vessels, and lymph nodes (Nathanson, 2003). The important roles of cell biomechanics in cancer invasion and metastasis potential have been documented (Denias et al., 2016; Emon et al., 2018; Gkretsi and Stylianopoulos, 2018). Most of the previous studies investigating the correlation of cancer metastasis with cell biomechanics focused mainly on the analysis of a single biomechanical factor, such as 2D cell random migration, cell traction force, or cell stiffness (Carey et al., 2012; Xu et al., 2012). However, in cancer metastasis, the invasion of cancer cells, through small pore sizes and micro-tunnels of surrounding 3D microenvironments, is orchestrated by a network of numerous biomechanical properties; cellular forces could enable the cancer cells not only to squeeze into narrow pores of 3D ECM but also to align the surrounding ECM fibers to form micro-tunnels to enhance the directional migration of cancer cells (Riching et al., 2014; Chen et al., 2019). Metastatic cancer cells are often softer with less cytoskeleton, compared with normal cells, resulting in higher invasion capability in trespassing the small pore size of microenvironments (Nathanson, 2003). Nuclear stiffness and morphology are also crucial factors that limit cancer cell invasion through the narrow pores of surrounding microenvironments (Fu et al., 2012; Wolf et al., 2013; Ribeiro et al., 2014). Moreover, the nuclear stiffness, which is approximately 2–10 times the corresponding value of the cytoplasm, has been regarded as a more important biomarker (in comparison with the cytoplasmic stiffness) in determining the invasion capability of cancer cells (McGregor et al., 2016). Hence, in this study, we suggested that all these biomechanical properties and behaviors of cancer cells should be systematically taken into account in the assessment of metastasis potential. In our work, to better mimic LN metastasis, we used mesenchymal-type HNSCC cell line SAS cells (of human HNSCC cells), to establish an LN-metastatic mouse model *in vivo* to scrutinize the LN-metastatic and non-metastatic HNSCC cells. By comparing the biomechanical features of LN-metastatic and non-metastatic HNSCC cells, we found that LN-metastatic cells managed to invade into the LNs, by increasing cell traction forces and decreasing the nuclear stiffness, thereby increasing their longitudinal strain. Our previous studies indicated that the cytoplasm of mesenchymal-type HNSCC cells was softer; i.e., it exhibited smaller value of elastic modulus than the epithelial-type HNSCC cells (Chen et al., 2018). Furthermore, we found no significant differences in cytoplasmic stiffness and distribution of actin filaments in LN vs. non-LN-metastatic HNSCC cells. Our current results implied that more pliable cell nuclei and greater cell traction force of LN-metastatic cells worked cohesively to strengthen their capability to invade through the small pores of the surrounding ECM or blood vessels.

Lamin A/C plays a critical role in regulating the nuclear stiffness and maintaining nuclear morphology, which has a significant impact on the invasion capability of cancer cells (Denais et al., 2016). Wang et al. (Wang et al., 2019) reported that when lamin A/C was dysfunctional, the invasion capability of ovarian cancer cells through narrow (3 μm) pores was reduced. The overexpression of lamin A/C caused stiffer nuclei, thereby disabling the cells to pass through the small pores; on the other hand, the cell nuclei, with the underexpression of lamin A/C, could become too soft and too fragile such that they broke into pieces during the invasion through the narrow pores.

**FIGURE 7:**
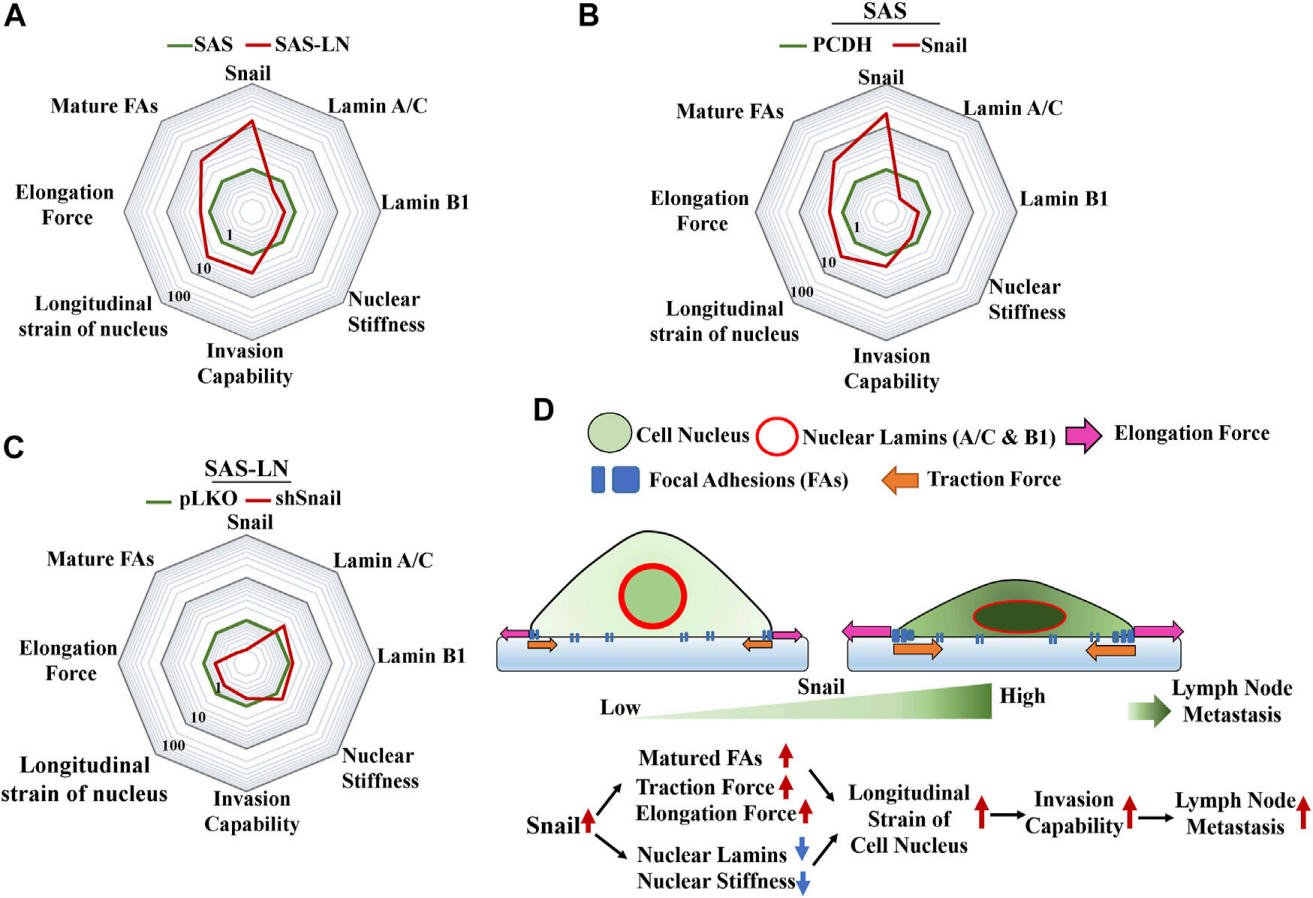
Summary of the changes in the biophysical and biochemical properties of HNSCC cells regulated by Snail for lymph node metastasis. A radar chart presents quantitatively the fold change in biophysical and biochemical properties in **(A)** SAS vs. SAS-LN cells, **(B)** SAS cells (SAS-PCDH) vs. Snail-overexpressed SAS cells (SAS-Snail), and **(C)** SAS-LN (SAS-LN-pLKO) vs. Snail-knockdown SAS-LN (SAS-LN-shSnail). The value of each parameter in the three radar charts was normalized by the corresponding values of SAS, SAS-PCDH, and SAS-LN-pLKO, respectively. **(D)** A schematic diagram to summarize the proposed model illustrating the effect of Snail on the biophysical and biochemical properties of HNSCC cells in lymph node metastasis.

Cancer invasion also requires dynamic alteration of cellular morphology and interaction with the surrounding ECM through dynamic assemblies and turnover FAs, which is stimulated by traction force or mechanical tension (Mierke et al., 2017). The FA transition from nascent adhesions to mature focal complexes contributes to membrane tension during migration or invasion (Tilghman & Parsons, 2008; Mierke et al., 2017). In this study, we further addressed the correlation between the LN metastasis and the nuclear strain. We found that the stronger traction force could be correlated with the elevated number of large focal adhesion-associated proteins, whereas lower nuclear stiffness could be correlated with the suppressed expression of nuclear lamin A/C and B1 (Figure 7A). These changes in biomolecular and biomechanical characteristics were indispensable in cancer metastasis. Importantly, these biomolecular and biomechanical features of LN-metastatic cells can be used as biomarkers to assess the gene regulation and the subsequent elevation of LN metastasis.

Li et al. (2019) revealed that knockdown of Snail had a great impact in reducing LN metastasis of SCC in a mouse model. However, the correlation between the Snail expression and biomechanical properties of LN-metastatic cells for LN metastasis has not been addressed. Here, we showed that Snail overexpression led to stronger traction force and lower nuclear stiffness of HNSCC cells with an associated increment in focal adhesion maturation and decrement in lamin A/C and B, to elevate the longitudinal strain of nuclei and promote cancer invasion capability (Figure 7B). Conversely, Snail knockdown in SAS-LN cells reduced the longitudinal strain of cell nuclei and cell invasion capability (Figure 7C). Here, the nuclear strain was determined by analyzing the length difference of the cell nuclei before and after trypsin treatment via nuclear stiffness assay, and invasion capability was determined by counting the number of cells invading into the 12 μm microchannels from the access ports. Based on these results, we have shown that Snail expression plays an important role in the promotion of LN metastasis by the enhancing longitudinal strain of cell nuclei, thereby enabling the cells to squeeze and invade into narrow pores of the tumor microenvironment (Figure 7D). Therefore, Snail-mediated intervention may be a potential therapeutic method for targeted manipulation of gene expression for cancer treatment.

## Data Availability

The original contributions presented in the study are included in the article/Supplementary Material, further inquiries can be directed to the corresponding author.
