# Supplementary material for: Snail Augments Nuclear Deformability to Promote Lymph Node Metastasis of Head and Neck Squamous Cell Carcinoma
Source: Front Cell Dev Biol. 2022 Feb 21;10:809738. doi: 10.3389/fcell.2022.809738 (PMC8899106; doi:10.3389/fcell.2022.809738)
Supplement: Supplementary file 1 [file DataSheet1.docx]

Supplementary Material

**
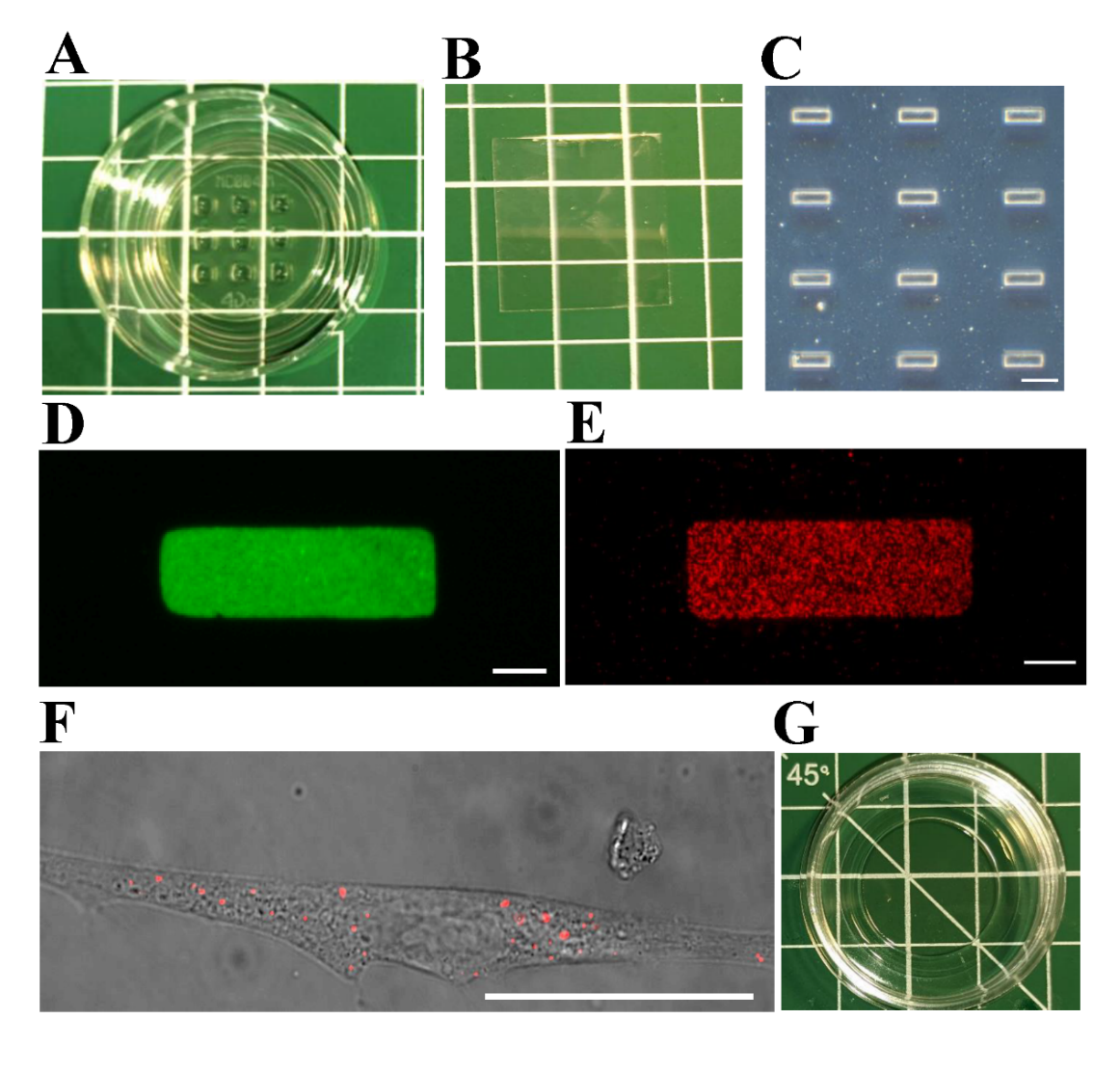
**

**Supplementary Figure 1. Pictures and Micrographs of experimental devices. (A)** A microchannel assay; white grids is 1cm x 1cm. **(B)** A nuclear stiffness assay consisting of rectangular micropattern and traction force assay, fabricated on a polyacrylamide coated glass coverslip (22mm x 22mm). **(C)** Phase contrast images of rectangular micropattern (length = 55 μm and width = 18 μm) array. Scale bar = 50 μm. **(D)** Fibronectin-coated adhesive rectangular islands on top of the polyacrylamide substrate; Green: FITC-labeled fibronectin. **(E)** 200 nm-diameter fluorescent beads were embedded in polyacrylamide substrate to serve as the probes for the quantification of cell traction forces on the substrate. Scale bar = 10μm. **(F)** For video particle tracking microrheology (VPTM), cells were injected with 200 nm-diameter carboxylated polystyrene particles via a biolistic particle delivery system and **(G)** cultured on 35 mm collagen coated glass bottom culture dishes (α-PLUS). Scale bar = 25 μm.

**
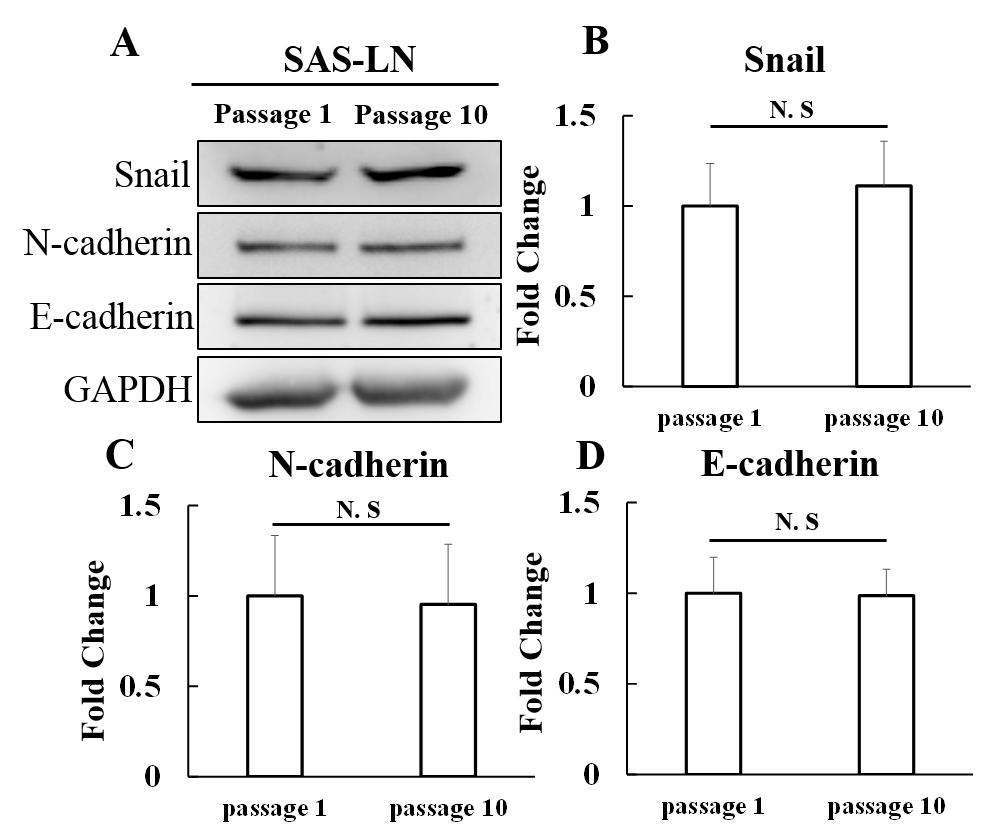
**

**Supplementary Figure 2. The expression of epithelial-mesenchymal transition markers of SAS-LN cells in passages 1 and 10 were similar.** GAPDH was used as loading control. **(A)** Western blot of Snail, N‐cadherin and E‐cadherin of SAS-LN cell in passages 1 and 10. Fold change expression of **(B)** Snail, **(C)** N‐cadherin, and **(D)** E‐cadherin relative to cell passage 1. Data represent mean ± SD (n = 3). N.S: Not Significant.

**
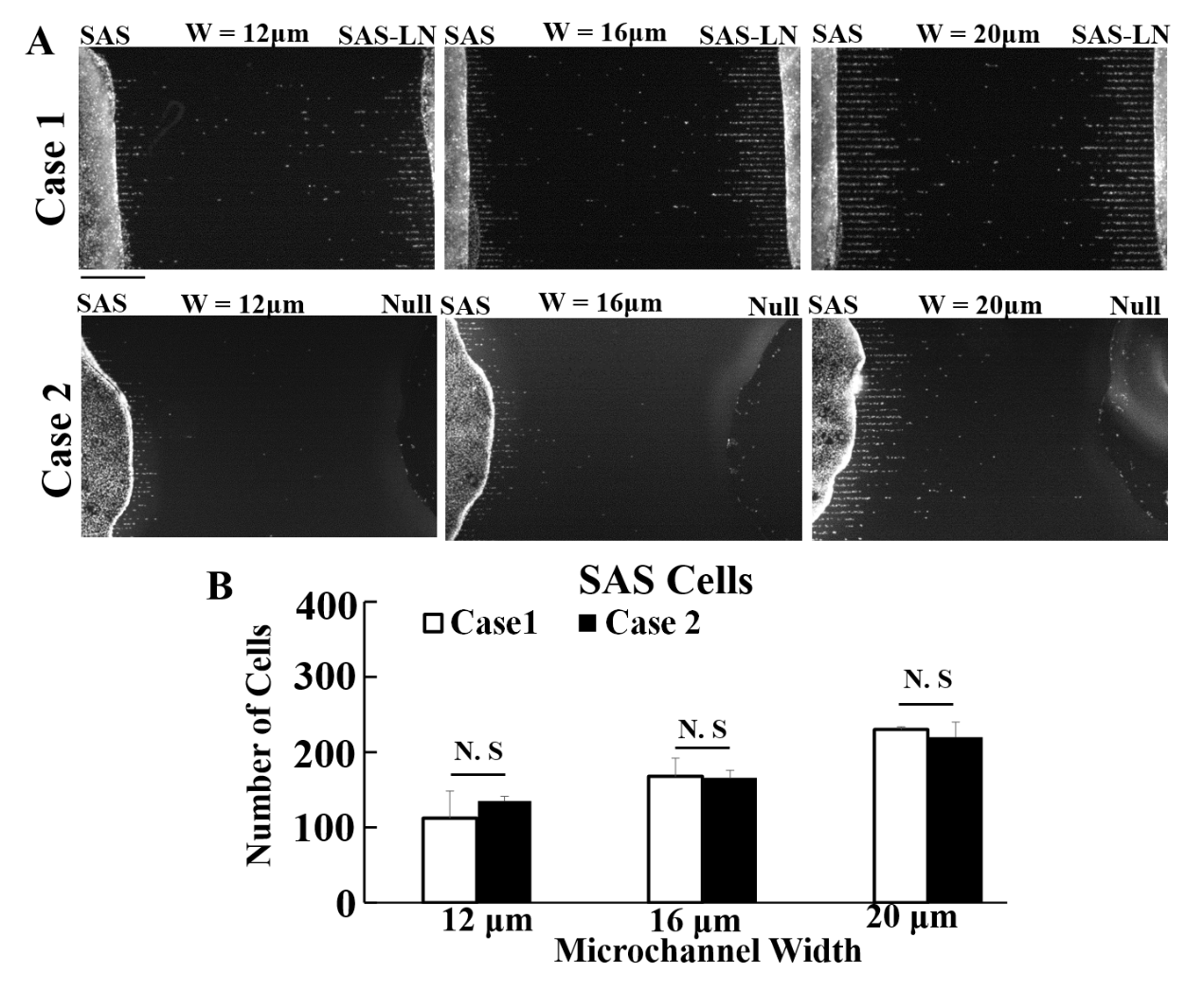
**

**Supplementary Figure 3.** **The numbers of SAS cells invaded from the access ports into the microchannels with vs. without seeding SAS-LN cells on the opposite side of access ports.** **(A)** To visualize and count the number of cells in the microchannels after seeding cells for 24hrs, the cell nuclei were stained with hoechst 33342. Scale bars = 400 μm. **(B)** Analysis of numbers of cells invaded into the microchannels. Data represent mean ± SD (n = 3). N.S: Not Significant.


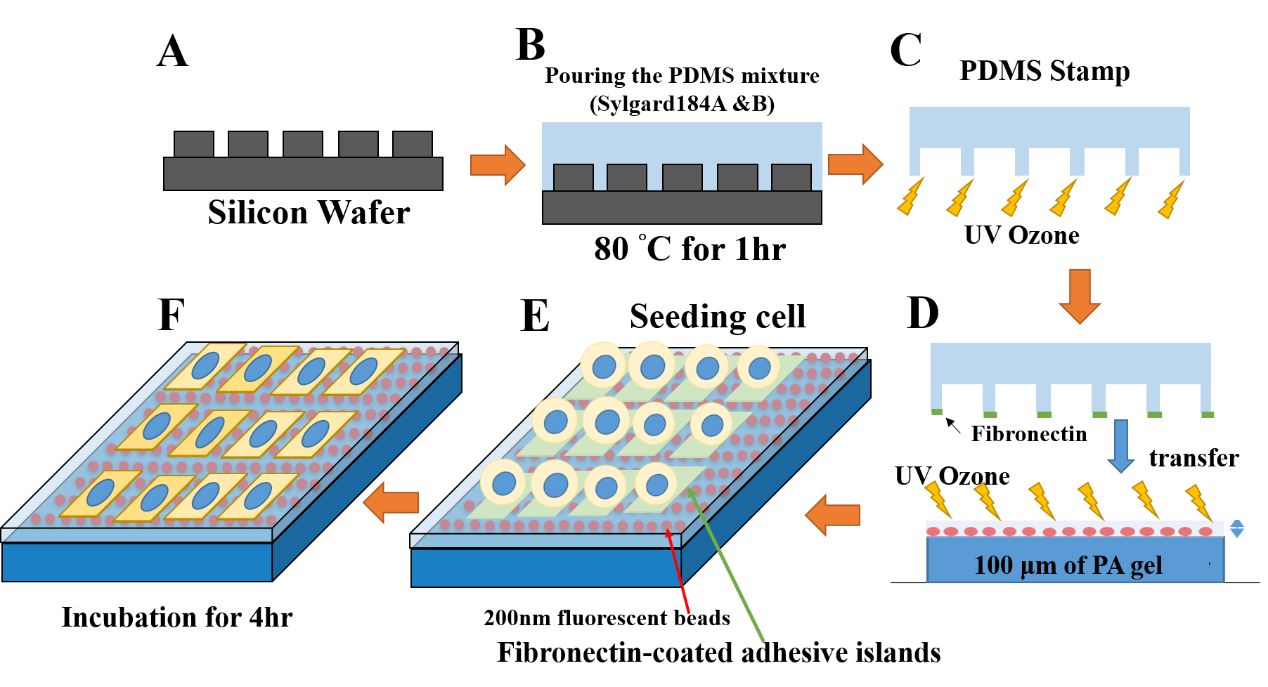


**Supplementary Figure 4. A schematic diagram of the fabrication procedure for micropattern based nuclear stiffness assay. (A)** A silicon wafer, bearing negative patterns of the designed rectangular micropatterns. **(B)** Pouring and spreading of the PDMS mixture (10:1 weight ratio of Sylgard184A and 184B) on silicon wafer and cure at 80 °C for 1hr. **(C)** A PDMS stamp treated with UV ozone for 2 min and soaked with 50 µg/ml of fibronectin solution for 5 min. **(D)** Fabrication of fibronectin-coated adhesive islands on top of the polyacrylamide substrate (mixing 0.1 % of Bis with 10% of acrylamide to obtain 9 kPa gels and mixed with 200 nm-diameter fluorescent beads). **(E)** & **(F)** seeding and incubation of cells on micropattern based nuclear stiffness assays.


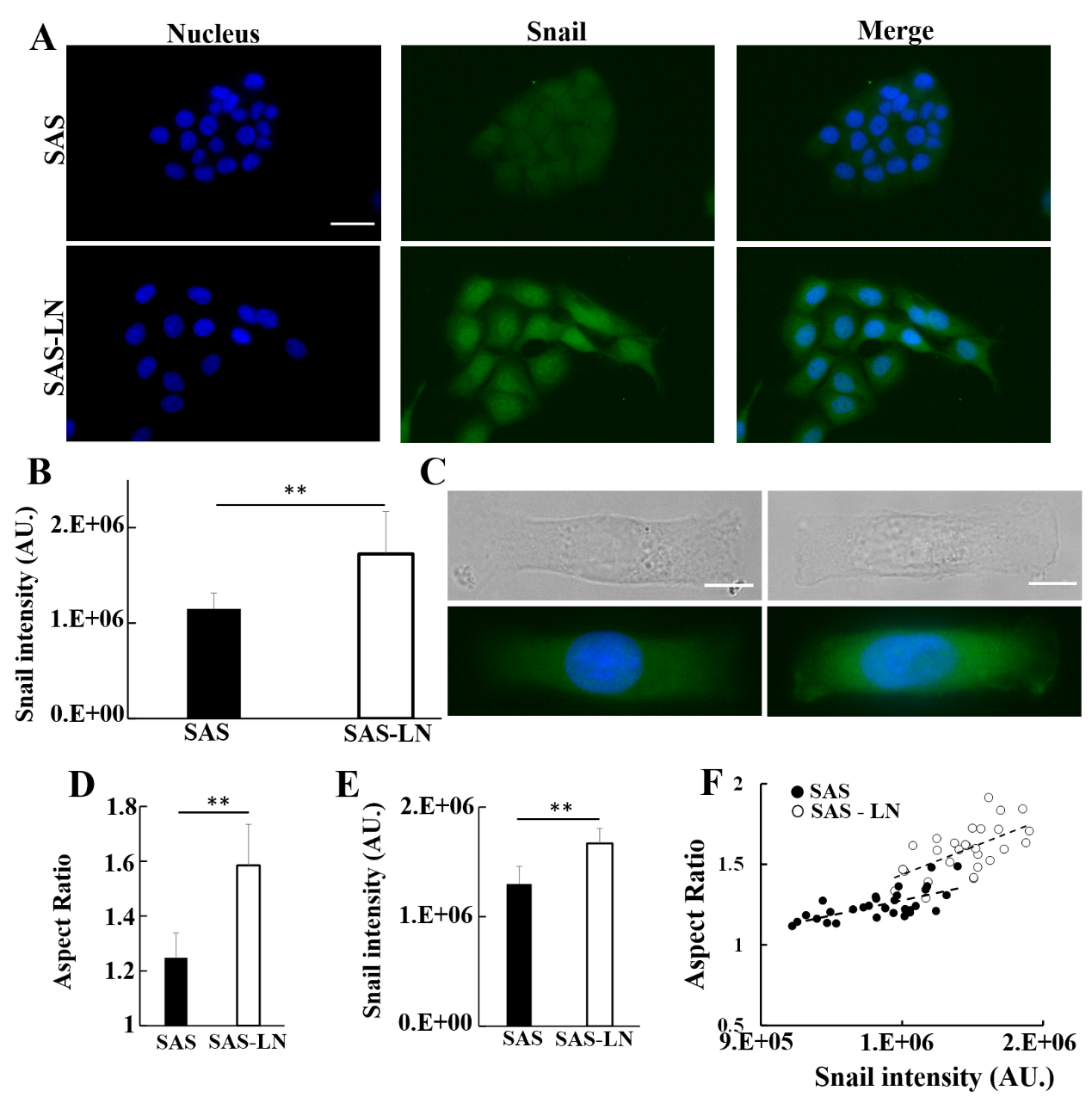


**Supplementary Figure 5.** **The correlation between Snail expression and elongation of cell nuclei.** **(A)** Immunofluorescence images of SAS-LN and SAS cells cultured on a glass-bottom dish. Blue: nucleus (Hoechst33342), Green: FITC-labeled Snail. Scale bars = 50 μm. **(B)** Analysis of integrated fluorescence intensity of Snail in SAS-LN and SAS cells. Data represent mean ± SD (n = 60). **(C)** Immunofluorescence and phase contrast images of SAS-LN and SAS cells, cultured on rectangular micropatterns (length = 55 μm and width = 18 μm). Scale bars = 10 μm. **(D)** Quantification of nuclear elongation in term of the aspect ratio ( i.e., major axis length / minor axis length of the cell nucleus). **(E)** Quantification of Snail intensity in SAS-LN and SAS cells, cultured on rectangular micropatterns. Data represent mean ± SD (n = 30). **(F)** A scatter plot of the snail expression vs. nuclear elongation of SAS and SAS-LN cells, cultured on rectangular micropatterns (n = 30).


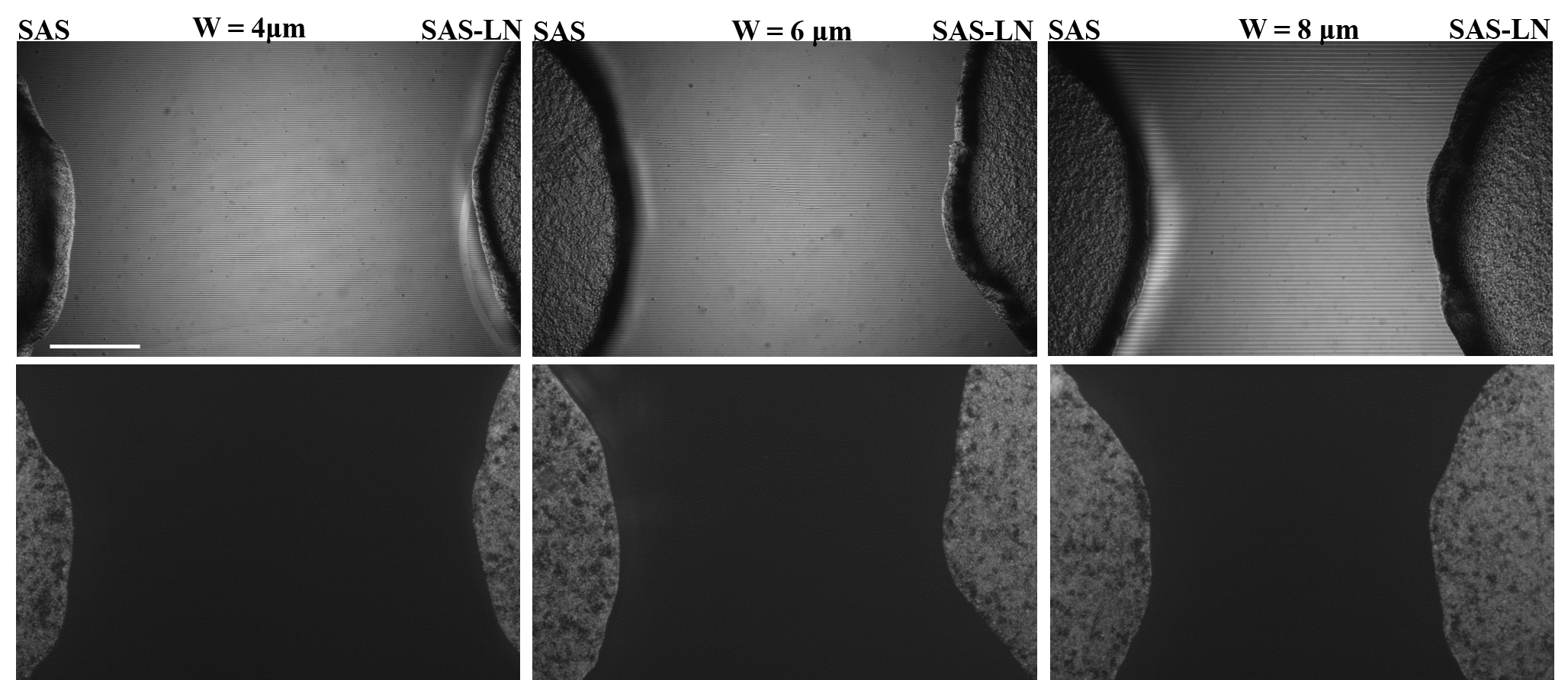


**Supplementary Figure 6. After seeded for 24 hrs, hardly any SAS-LN and SAS cells invaded from the access ports into the microchannels with channel widths below 8 μm.** Upper panels: the phase contrast images of cells in microchannels. Lower panels: the cell nuclei were stained with hoechst 33342 to visualize and count the number of cells in the microchannels. Scale bars = 400 μm.


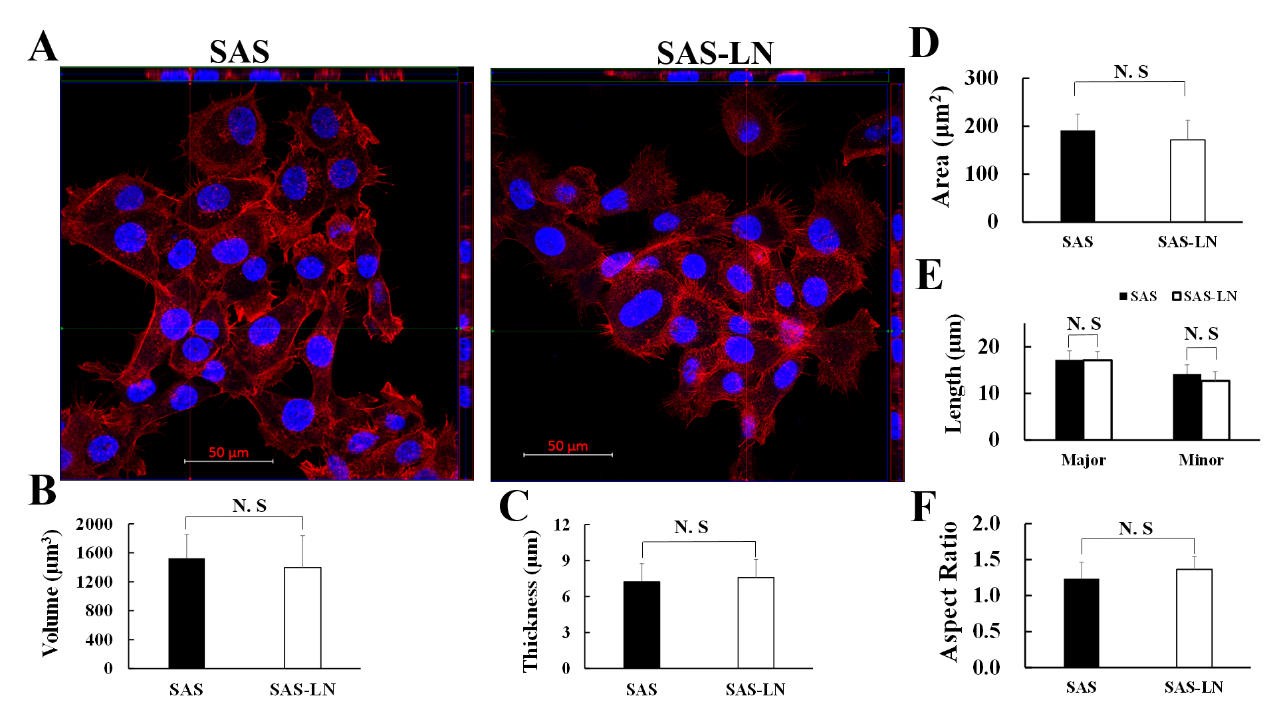


**Supplementary Figure 7.** **Nuclear morphology analysis of LN-metastatic cells (LN-SAS) and non-metastatic cells (SAS) cultured on a glass-bottom dish.** **(A)** Representative immunofluorescence images of SAS-LN and SAS cells. Blue: nucleus (Hoechst33342), Red: TRITC-labeled phalloidin for actin. **(B-F)** Quantification of nuclear morphology of SAS-LN cells and SAS cells, in terms of volume, thickness, area, length, and aspect ratio. Data represent mean ± SD (n =20).


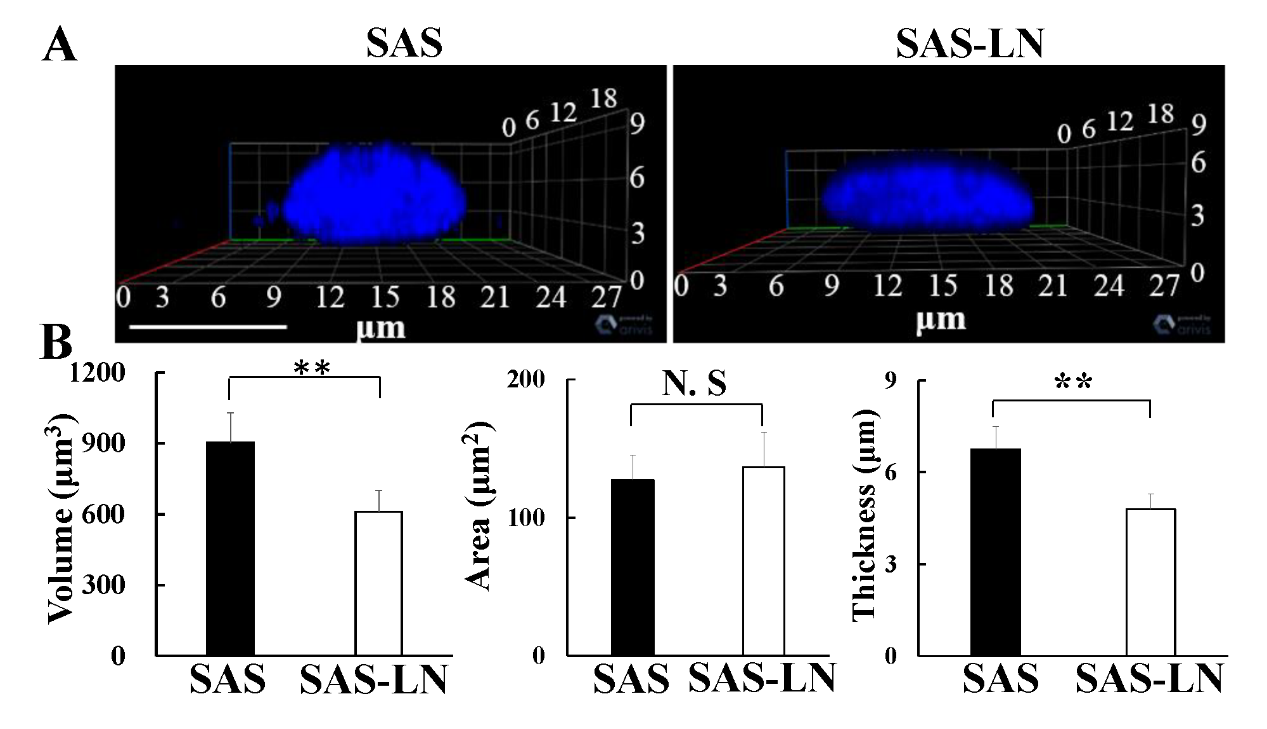


**Supplementary Figure 8. Characterization of nuclear morphology of LN-metastatic HNSCC cells (SAS-LN) and non-metastatic HNSCC cells (SAS). (A)** The reconstituted 3D confocal micrographs of cell nucleus. Blue: cell nucleus (Hoechst33342). Scale bars = 9 μm. **(B)** Analysis of nuclear morphology of SAS-LN and SAS cells, cultured on rectangular micropatterns (length = 55 μm and width = 18 μm). Data represent mean ± SD (n = 20). * for p < 0.05 and ** for p < 0.01; N.S: Not Significant.


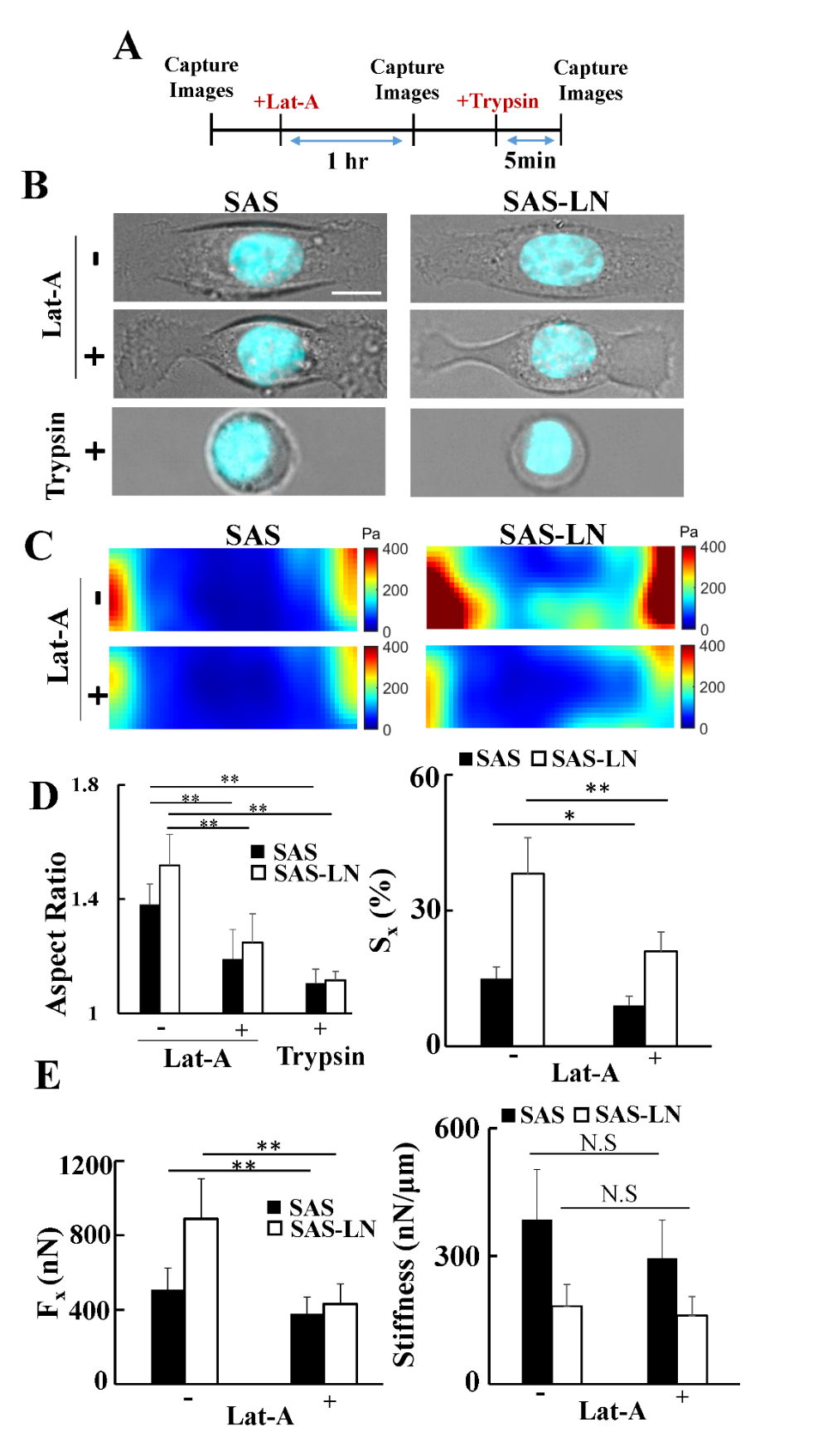


**Supplementary Figure 9. The inhibition of actin polymerization reduces cellular forces and nuclear deformation. (A)** A schematic diagram of the experimental procedure. **(B)** Phase contrast and fluorescent images of nuclear morphology of SAS-LN cells and SAS cells, cultured on the rectangular micropatterns, before (upper panels) and after (middle panels) treatment of 0.2μM latrunculin A (Lat-A) for 1 hr, and (lower panels) trypsin treatment for 1 hr. Blue: cell nucleus. Scale bars = 10 μm. **(C)** Representative images showing 2D distributions of the magnitude of traction forces in SAS-LN cells and SAS cells before (upper panels) and after (lower panels) treatment of latrunculin A (0.2μM). **(D)** Left: Comparison of the aspect ratio of nuclei morphology of SAS-LN cells and SAS cells before and after treatment of latrunculin A (0.2μM). Right: Analysis of the longitudinal strain (S_x_) of cell nuclei before and after trypsin treatment. **(E)** Analysis of elongation forces (F_x_) and nuclear stiffness of SAS-LN cells and SAS cells before and after treatment of Lat-A. Data represent mean ± SD (n = 20).


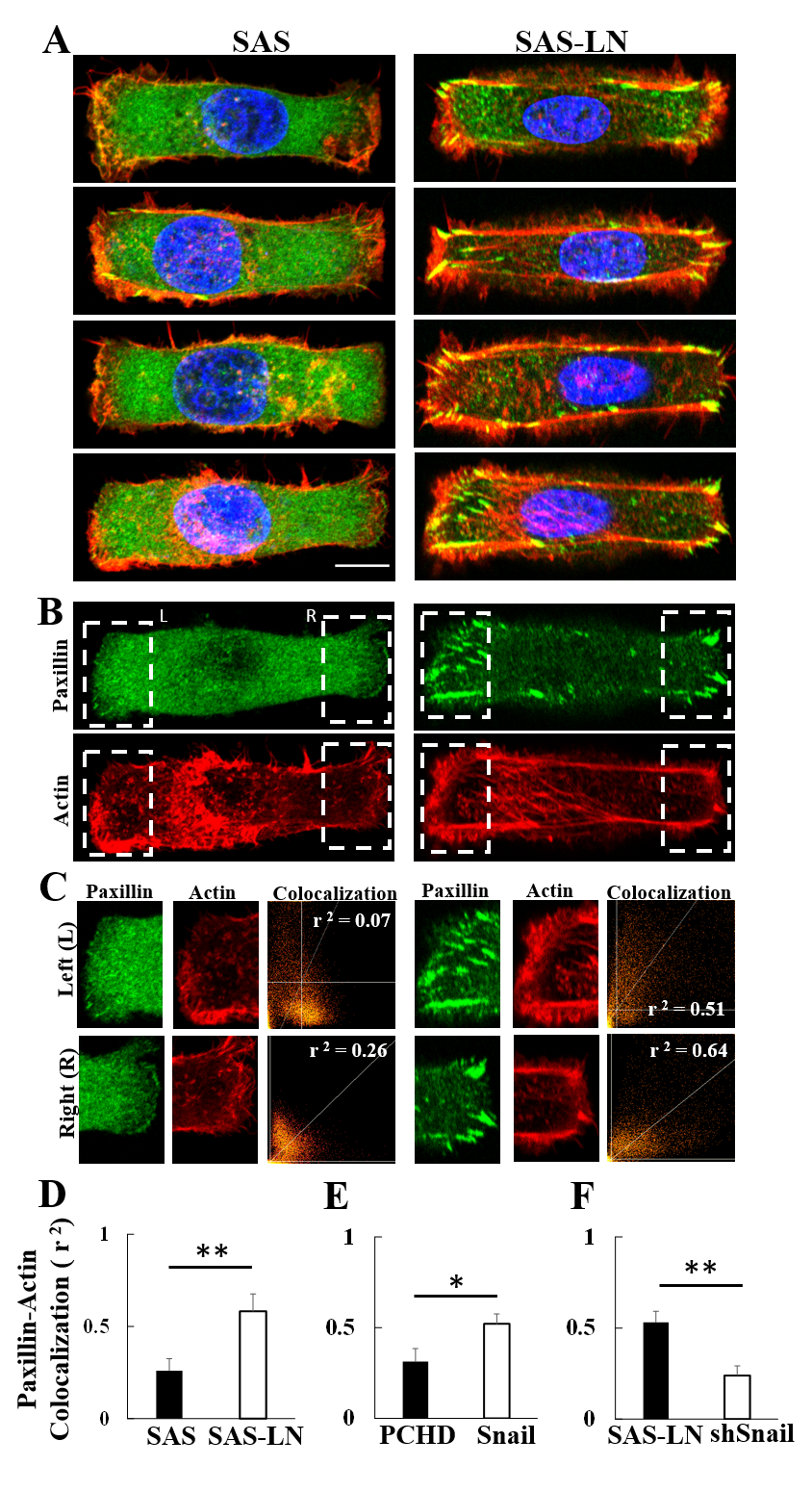


**Supplementary Figure 10.** The analysis of co-localization of paxillin and actin filaments at the edges of the elongated cells. **(A)** Random selection of 4 examples for the immunofluorescence micrographs of focal adhesion proteins paxillin and actin filaments in SAS and SAS-LN cells. Scale bars = 10 μm. **(B)** Magnified images of paxillin (left panel) and actin filaments (middle panel) in the white square regions, 20 μm × 12 μm each, as shown in (A); **(C)** right panel: A scatter plot of paxillin vs. actin filaments; the degree of co-localization of paxillin and actin filaments was quantified by Pearson’s correlation coefficient (r^2^). A comparison of the measure of co-localization of paxillin and actin filaments in **(D)** SAS vs. SAS-LN cells, **(E**) SAS cells (SAS-PCDH) vs. snail-overexpressed SAS cells (SAS-Snail), and **(F)** SAS-LN (SAS-LN-pLKO) vs. Snail-knockdown SAS-LN (SAS-LN-shSnail). All data are expressed as mean ± SD from 15 cells.


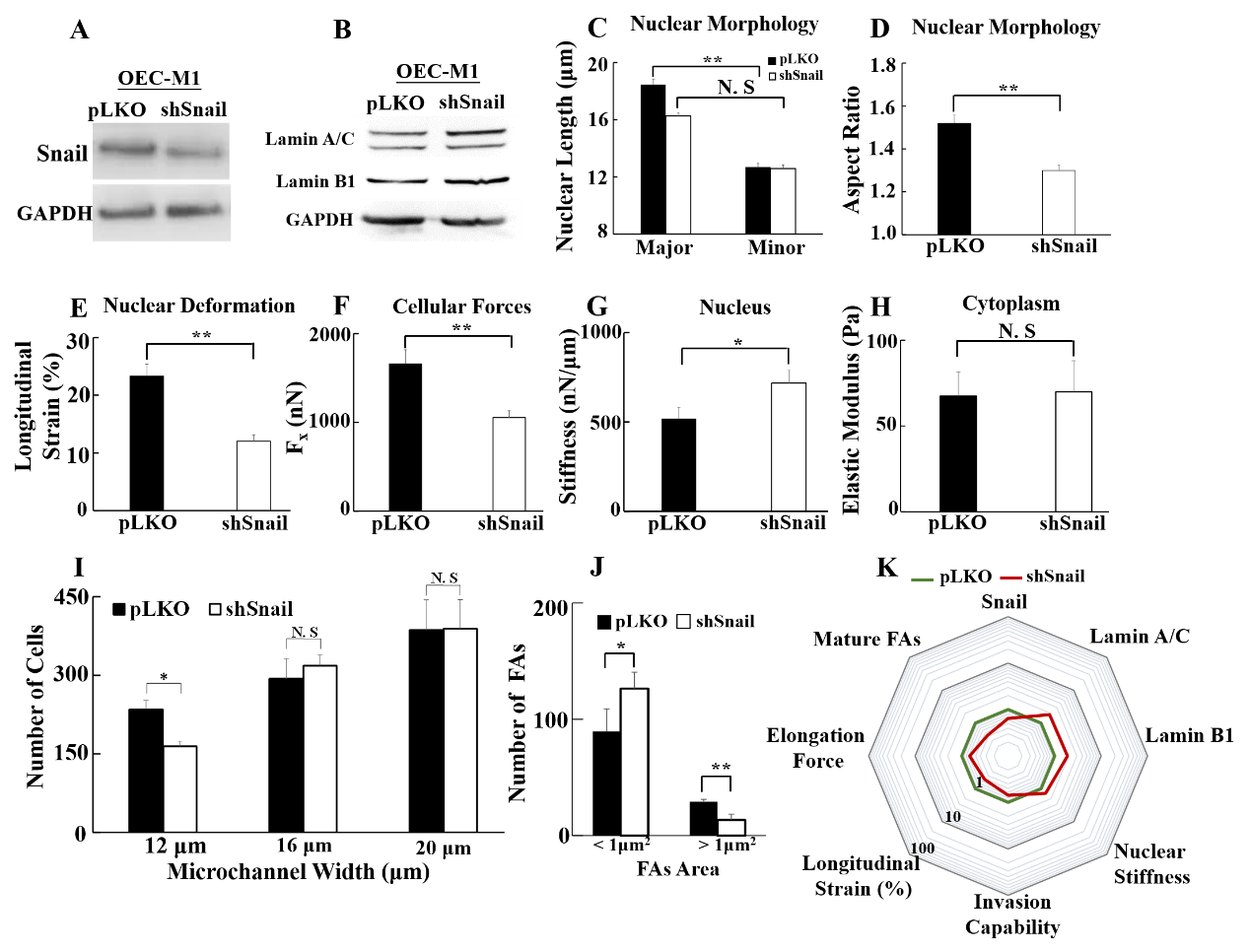


**Supplementary Figure 11.** **The effect of Snail knockdown on biomolecular and biomechanical properties of OEC-M1 cells.** **(A)** Western blot of Snail in control (OEC-M1-pLKO) using pLKO-shCtrl and Snail1-knockdown OEC-M1 (OEC-M1-shSnail) using pLKO-shSnail, with GAPDH as a loading control. **(B)** Knockdown of Snail enhanced the expression of lamin A/C and B1. **(C-D)** Knockdown of snail induced a round morphology of cell nuclei. **(E-H)** Knockdown of snail reduced longitudinal strain of nuclei and elongation forces, enhanced nuclear stiffness, but rendered smaller effect in cytoplasmic stiffness. Data represent mean ± SD (n =20). **(I)** Numbers of OEC-M1-shSnail and OEC-M1-pLKO cells invaded for 24hrs from the access ports into the microchannels (with different widths of 12, 16, and 20 μm). Data represent mean ± SD (n = 3). **(J)** Knockdown of snail reduced the amount of mature FAs (FAs area > 1 μm^2^). **(K)** A radar chart to present quantitatively the fold change in biophysical and biochemical properties in OEC-M1-shSnail vs. OEC-M1-pLKO cells.
